# The Sonar Model for Humpback Whale Song Revised

**DOI:** 10.3389/fpsyg.2018.01156

**Published:** 2018-07-16

**Authors:** Eduardo Mercado III

**Affiliations:** ^1^Department of Psychology, University at Buffalo, The State University of New York, Buffalo, NY, United States; ^2^Evolution, Ecology, and Behavior Program, University at Buffalo, The State University of New York, Buffalo, NY, United States

**Keywords:** auditory enhancement, auditory scene analysis, bioacoustics, biosonar, cetacean, echolocation, singing, spatial hearing

## Abstract

Why do humpback whales sing? This paper considers the hypothesis that humpback whales may use song for long range sonar. Given the vocal and social behavior of humpback whales, in several cases it is not apparent how they monitor the movements of distant whales or prey concentrations. Unless distant animals produce sounds, humpback whales are unlikely to be aware of their presence or actions. Some field observations are strongly suggestive of the use of song as sonar. Humpback whales sometimes stop singing and then rapidly approach distant whales in cases where sound production by those whales is not apparent, and singers sometimes alternately sing and swim while attempting to intercept another whale that is swimming evasively. In the evolutionary development of modern cetaceans, perceptual mechanisms have shifted from reliance on visual scanning to the active generation and monitoring of echoes. It is hypothesized that as the size and distance of relevant events increased, humpback whales developed adaptive specializations for long-distance echolocation. Differences between use of songs by humpback whales and use of sonar by other echolocating species are discussed, as are similarities between bat echolocation and singing by humpback whales. Singing humpback whales are known to emit sounds intense enough to generate echoes at long ranges, and to flexibly control the timing and qualities of produced sounds. The major problem for the hypothesis is the lack of recordings of echoes from other whales arriving at singers immediately before they initiate actions related to those whales. An earlier model of echoic processing by singing humpback whales is here revised to incorporate recent discoveries. According to the revised model, both direct echoes from targets and modulations in song-generated reverberation can provide singers with information that can help them make decisions about future actions related to mating, traveling, and foraging. The model identifies acoustic and structural features produced by singing humpback whales that may facilitate a singer’s ability to interpret changes in echoic scenes and suggests that interactive signal coordination by singing whales may help them to avoid mutual interference. Specific, testable predictions of the model are presented.

## Introduction

Humpback whales (*Megaptera novaeangliae*) use sound more flexibly than most terrestrial mammals (Herman and Tavolga, 1980; Mercado et al., 2014). Some regard the “songs” of humpback whales, in particular, as the most sophisticated acoustic displays in the animal kingdom (Wilson, 1975; Winn and Winn, 1978). Fifty years of field research have established that humpback whale songs play a critical role in the mating system of humpback whales (for reviews, see Helweg et al., 1992; Parsons et al., 2008; Herman, 2017). Researchers have debated about exactly how humpback whales use songs to facilitate sexual behavior, but there is near-universal agreement that humpback whale songs are sexual signals that male humpback whales use in an attempt to increase their mating opportunities. The hypothesis that songs serve primarily as reproductive displays has dominated since the first scientific reports that humpback whales sing (Winn et al., 1970; Payne and McVay, 1971). Here, it is argued that much of the observational, experimental, and comparative evidence collected to date provides little support for sexual advertisement hypotheses, and that current data are better accounted for by the alternative hypothesis that humpback whales “sing” primarily to actively explore the world around them.

## Song as Sexual Advertisement

The current scientific consensus regarding the nature of humpback whale song is: (1) songs are part of a sophisticated acoustic communication system; (2) songs can be heard several kilometers away from a singer; (3) singers can repeat songs continuously for multiple hours; (4) songs are unique among cetaceans and other mammals in terms of their complexity; (5) the complexity of songs comes from their hierarchical structure and the fact that song features change over time; and (6) singers within a population converge on similar songs through a process of cultural transmission (Sardelis, 2017). Three proposals for reproductive functions of songs are that they provide a way for females to find high quality males, mediate long-distance male-male competition or affiliation, and/or attract females from long distances (Herman, 2017). Descriptions of songs, singer behavior, the behavior of other whales exposed to song, and the behavior of other singing species, have been cited as support for each of these reproductive functions. Probably the most compelling evidence supporting claims that songs are sexual advertisements are reports that humpback whales sing predominately during the breeding season and that singers are exclusively males.

Humpback whales sing in various behavioral contexts, leading some to propose that songs serve multiple functions (e.g., Dunlop and Noad, 2016). Correlations between the spacing of singers and the density of whales in a region suggest that songs may function as intra-sexual spacing signals (Frankel et al., 1995; Seger et al., 2016). Singers sometimes accompany females with calves, which has been interpreted as evidence that songs function as courtship displays (Smith et al., 2008). Regions where multiple singers are audible could function as acoustic lekking arenas that attract females (Herman, 2017). Each of these proposals is derived from communicative mating displays identified in other species. Key variables used to support such generalizations to whales include: distances between senders and receivers, the sex, size, and reproductive status of singers and listeners, approach and avoidance behavior in different social groups, the performance of acts associated with copulation during or after social interactions related to singing, and the initiation and cessation of singing. Cross-species comparisons of vocal behavior are critical to gaining a clear understanding of why humpback whales sing and why their songs are so dynamic.

### Comparisons With Singing by Birds

From the earliest reports of singing by humpback whales, researchers have compared their songs to birdsongs, noting similarities in the diversity of sounds, the regularity of repeated patterns within songs, their similar hierarchical structures, and their possible roles in mating. Sped-up playbacks of humpback whale song subjectively sound like singing birds, and slowed-down playbacks of birdsong are reminiscent of singing whales (Rothenberg, 2014). Winn and Winn (1978) noted several structural features of songs that make them suitable for long-distance communication, including their length, monotony, redundancy, diversity of frequency content, and rhythmicity, most of which are also evident in birdsongs (Catchpole and Slater, 2008).

Evidence that humpback whales progressively change their songs led researchers to begin comparing this phenomenon to song learning, vocal repertoire expansion, and cultural transmission by songbirds (Tyack, 1981). Such comparisons remain prevalent. For instance, Garland et al. (2017) suggested that song learning by humpback whales is most similar to song learning by orange-tufted sunbirds (*Nectarina osea*), village indigobirds (*Vidua chalybeate*), yellow-rumped caciques (*Cacicus cela vitellinus*), and black-capped chickadees (*Poecile atricapillus*).

The ever-changing properties of humpback whale songs, combined with cross-population differences in song content, suggest that singers are not only adept at learning songs, but also have sophisticated improvisational skills (Payne, 2000; Parsons et al., 2008). Innovations can potentially increase the diversity and complexity of a song. In songbirds, such changes can increase a song’s effectiveness as a mating display (Catchpole, 1987). Similarly, innovative humpback whale singers might gain reproductive benefits.

Perceptual similarities, structural similarities, and parallels in vocal repertoire modification provide more than sufficient grounds for describing the sound sequences produced by humpback whales as songs comparable to those produced by birds. It is ultimately similarities in the ecological contexts within which songs are produced, however, that have convinced scientists that humpback whale songs function like bird songs. Given that in several species of birds only the males sing, and that they do so primarily or exclusively during breeding seasons, it stands to reason that if only male humpback whales sing, and do so mainly during breeding seasons, then they might be singing for similar reasons as birds—namely to attract and court females and/or to repel male competitors. Singing humpback whales do space themselves out on the breeding grounds (Frankel et al., 1995), supporting the prediction that songs can repel other singers.

If humpback whales sing for the same reasons as birds, then this could explain why humpback whales produce such extravagant songs. Darwin (1871) proposed that the musical qualities of birdsong evolved because females preferentially mated with males that sang the most beautiful songs, an evolutionary process he termed sexual selection. Female preferences for novelty, complexity, or song length could have led to the evolution of elaborate structural and dynamic complexity within humpback whale songs (Payne, 2000; Parsons et al., 2008). Such preferences might arise from associations between song properties and male quality, female sensory biases, or positive feedback between female preferences and embellishments of sexual signals. Female preferences might also explain why humpback whales sometimes sing continuously for several hours. Prolonged singing by songbirds that does not involve counter-singing between males is thought to function primarily as a female attractant (Catchpole, 1987).

Any commonalities between the songs and singing behavior of humpback whales and songbirds might suggest convergent evolution of sexual signals in these taxa, predicting that additional similarities between these two groups might be found. For instance, songbirds possess specialized neural circuitry for learning, recognizing, and producing songs. Humpback whales might similarly be expected to show specializations in the neural control and processing of songs (Wright and Walsh, 2010). The ability of birds and humpback whales to rapidly adopt and evaluate new songs over time may depend on similar learning mechanisms, which could lead to similarities in learning trajectories (Garland et al., 2017), or in the use of rhythm and repetition (Guinee and Payne, 1988; Gray et al., 2001; Handel et al., 2012).

### Are Humpback Whale Songs Sexual Advertisement Displays?

Sexual advertisement hypotheses have been a powerful positive force in field studies of humpback whale behavior and in past analyses of song structure. However, the ability of these hypotheses to predict and account for empirical findings has diminished over time, even as the variety of communicative functions attributed to humpback whale songs has steadily increased. Additionally, many of the apparent similarities between singing birds and singing humpback whales noted above raise more questions than they answer about how songs function.

Evolutionary and ecological factors suggest that humpback whales are unlikely to have evolved acoustic behavior or mating systems comparable to those of songbirds because the ancestors of humpback whales faced vastly different environmental constraints, social systems, and developmental conditions from those encountered by songbirds. Unlike humpback whales, most songbirds: (1) sing in air during the day rather than underwater during both day and night; (2) are raised by parents (from whom they typically learn songs); (3) live in forests, where they establish and defend territories; (4) pair bond with mates; and (5) form relatively long-term social relationships with their neighbors, including dominance hierarchies. Sexual advertisement hypotheses assume that the acoustic behavior of humpback whales has diverged substantially from that of other cetaceans. But, why humpback whales would diverge so dramatically from close relatives that faced similar environmental and perceptual challenges, while converging evolutionarily with much more distant relatives with whom they share few environmental or social constraints is unclear. Runaway sexual selection is often mentioned by proponents of sexual advertisement hypotheses as a possible explanation for why humpback whale evolution diverged from that of other cetaceans and converged with bird evolution. However, other species that have evolved extravagant secondary sexual characteristics typically show progressive increases in the size or complexity of those characteristics across the lifespan (Manning, 1985), whereas changes to song features made by humpback whales do not accumulate (Payne and Payne, 1985). Possibly, female humpbacks could judge singers based on their improvisational or imitative skills (Parsons et al., 2008), instead of evaluating variations in song structure and diversity the way that songbirds do.

For sexual selection to drive evolution of an acoustic display, females must be able to discriminate and choose mates based on display features that are correlated with male fitness. In songbirds, intrasexual selection is evidenced by experiments showing that songs affect the outcomes of male contests and that mating success is correlated with these outcomes, while intersexual selection is indicated when females respond preferentially to particular song features, in the absence of males, that are correlated with male mating success (Searcy and Andersson, 1986). Given that there is currently no evidence of female humpback whales responding differentially to songs, or of songs affecting the outcomes of competitions between male humpback whales, claims that humpback songs are sexually selected displays must be considered speculative. Whether features within received songs enable listening whales to reliably distinguish physical or cognitive qualities of singers is unclear given both the distorting effects of sound propagation over long distances in humpback whale habitats (Mercado and Frazer, 1999), and the constantly changing acoustic properties of songs. Experimental playback studies are, of course, more difficult with humpback whales. Nevertheless, it is notable that when such experiments have been attempted, the findings have been the opposite of what sexual advertisement hypotheses predict—female humpback whales ignore or avoid speakers broadcasting songs, while male humpback whales commonly approach them (Mobley et al., 1988; Darling et al., 2012).

An early indication that humpback whale songs might be sexual advertisement displays was the fact that singing was most prevalent when females were most likely to conceive (Winn and Winn, 1978). This correlation provides circumstantial evidence that songs contribute to mating, but only weak evidence regarding what role songs may play. Recent large-scale efforts to monitor singing during migration (Garland et al., 2011) and on higher latitude feeding grounds (Watkins et al., 2000; Clark and Clapham, 2004; Vu et al., 2012; Garland et al., 2013; Stanistreet et al., 2013; Gong et al., 2014; Español-Jiménez and van der Schaar, 2018; Kowarski et al., 2018) show that singing is much less seasonally and geographically restricted than was originally thought. Humpback whales sing in every region where they can be found. Although concentrations of singers are highest in certain areas during the breeding season, the number of singers at these locations may be similar to the number that sing during migration or feeding. Song sessions are likely to be longer during the breeding season (Kowarski et al., 2018), which may further contribute to the impression that more whales are singing at this time.

Subjective similarities between birdsongs and sped-up humpback songs disguise numerous ways in which their songs differ. Humpback whales often sing continuously for hours without any clear interruptions that might indicate where one song ends and another begins, leading to debates about what sequences constitute a song. Singers produce individual sounds (called “units”) in predictable patterns (called “phrases”), which they often repeat multiple times. Sets of consecutively repeated phrases are called “themes”; this term is also sometimes applied to a non-repeated phrase that singers consistently produce. Singers produce multiple themes, often in a stereotyped order. Whale researchers traditionally define a “song” to be one cycle of ordered themes (Payne and McVay, 1971), but some argue that repeated phrases are more comparable to bouts of birdsongs (Cholewiak et al., 2013). Individual themes, as well as entire song sessions, would also qualify as “songs” based on current definitions (Spector, 1994). These ambiguities complicate cross-species comparisons and raise questions about why humpback whales evolved a song form so different from those used by songbirds—Winn and Winn (1978) noted that songbirds rarely cycle through their repertoire of songs in a fixed order. If singing whales are producing complex songs to compete for females, then it is surprising that they rarely vary their sequencing of phrases or themes given that this strategy, which is commonly used by songbirds, could increase both the novelty and complexity of their songs.

The progressive changes that humpback whales make to songs are often compared to song learning in birds, despite the fact that no species of bird (or any non-cetacean) shows such dramatic changes in sound patterning at the population level (Payne and Payne, 1985). Some songbirds (e.g., caciques) do gradually change elements of their songs over time, but the changes they make are trivial compared to those made by humpback whales, mainly involving subtle changes in a few sounds (Feekes, 1982). Furthermore, changes in birdsong repertoires, other than the addition of new songs, typically occur only after new members join a group. Even then, not all birds change their songs. In one study of village indigo birds, a species often compared to humpback whales, 76% of males did not change their songs to match a new variant (Payne, 1985). Finally, when songbirds do change their songs, the songs continue to be highly stereotyped in either their syntax or phonology—theories of long-distance communication suggest that such stereotypy is key to insuring that signals remain recognizable to listeners after long-distance propagation (Green and Marler, 1979). Importantly, this is a rule that singing humpback whales fail to follow.

**FIGURE 1:**
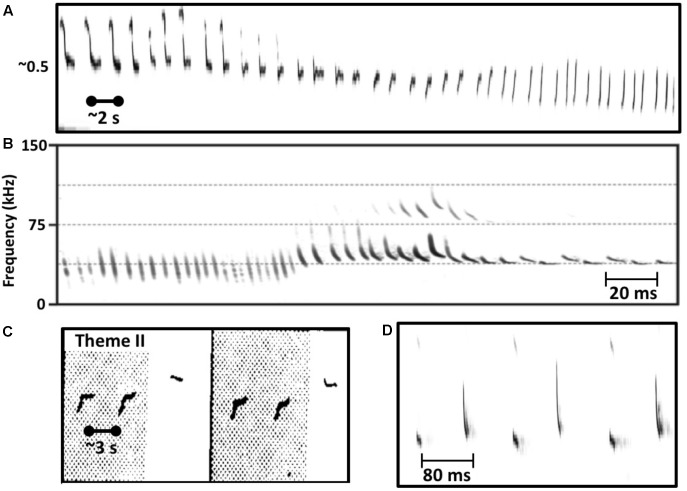
Similarities in the vocal behavior of singing humpback whales and echolocating bats. **(A)** Humpback whales use a graded repertoire of units. They sometimes gradually morph a unit with each repetition such that units early in the series are acoustically dissimilar from later units (spectrogram trace from Guinee and Payne, 1988, Figure 2). **(B)** Echolocating bats also use a graded repertoire, and also sometimes gradually morph signal features over time (from Mora et al., 2011, Figure 3). **(C)** Singing humpback whales produce regularly timed patterns of alternating units (spectrogram trace from Guinee and Payne, 1988, Figure 3). **(D)** Some bat species also produce rhythmically alternating sounds when echolocating (from Mora et al., 2011, Figure 2).

Perhaps the biggest difference between singing birds and singing humpback whales is that humpback whales continuously modify the individual sounds that they use within songs (Payne and Payne, 1985; Cato, 1991; Mercado et al., 2005). Singers gradually transform the tonality, duration, frequency contours, and even frequency ranges of units so extensively that prototypical units from a particular year are likely to be absent in songs recorded 5 years later. Even within a single bout of singing, a whale may gradually change acoustic features of units through repetition such that units late in the sequence contain no common acoustic features with units produced earlier in the sequence (**Figure 1A**), a case of natural *sound morphing* (Caetano and Rodet, 2013). The sound repertoire used by singing humpback whales is both graded and dynamic. Most mammals that use a graded repertoire of sounds to communicate do so only at close ranges where visual cues can reduce signal ambiguity (Green and Marler, 1979). It is thus quite surprising that humpback whale singers, who ostensibly are using songs to communicative over kilometer distances in an underwater environment that is highly prone to signal distortion (Mercado and Frazer, 1999), would vary their sounds so extensively. Such variation should make it difficult for whales listening from long distances to know whether the features of songs that they receive were produced by the singer or by transmission-related distortion. This property of humpback whale songs is especially puzzling if songs function as sexual advertisements, because the elements of every received song, even those produced by an individual singer, will differ depending on when and where the receiver hears them, confounding any cross-singer comparisons of song qualities.

## Song as Sonar

**FIGURE 2:**
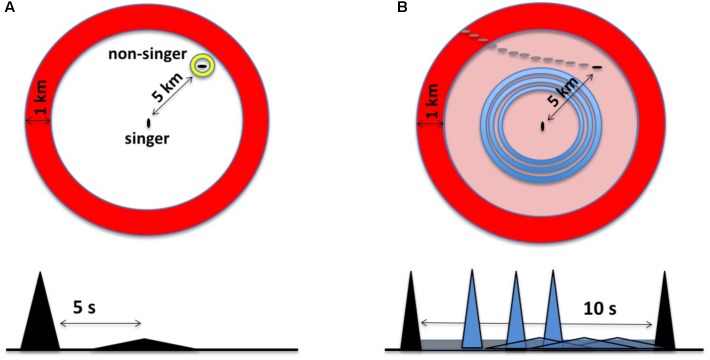
**(A)** The original sonar model characterized singing humpback whales as listening for a discrete echo from each unit produced. In the time domain (bottom), this involves recognizing a delayed and distorted replica of the unit. In this example, an echo from a whale located ∼5 km from the singer, represented as a flattened triangle, returns about 5 s after unit production ends. Spatially (top), echo processing involves perceiving the bearing and distance of the echo source within a two-dimensional, circular search space; the large annulus, or “unit ring,” corresponds to a snapshot in time of the spatial extent of a unit given its duration. **(B)** In the revised sonar model (the duplex model), singers generate multiple echo streams in parallel, integrating relative changes in both narrowband background reverberation and discrete broadband echoes to perceive the trajectories of moving echo sources. In the time domain (bottom), this involves listening for anomalies within familiar reverberation from CF-units (gray band), maximizing source detection, while simultaneously attempting to perceive the trajectory of a source using echoes from FM/broadband units (gray triangles). In the revised model, singers focus on detecting changes over time within their search space using CF units (outer ring) and FM units (inner rings).

Winn and Winn (1978) noted early on that echoes generated by humpback whale songs might provide singers with information about their distance from the surface or their proximity to nearby pinnacles or banks. These possibilities were never experimentally investigated; subsequent research has focused mainly on possible reproductive functions of songs. Frazer and Mercado (2000) hypothesized that biosonar is the primary function of humpback whale songs and developed a quantitative model to assess how songs might function as sonar. The model focuses on describing what happens each time a singing humpback whale makes a sound, and specifically on evaluating what units would be reflected by whale-sized targets, as well as how far song-generated echoes might travel before becoming undetectable. In brief, a singer generates an expanding annular acoustic field whenever it produces a unit (**Figure 2A**). The “unit ring” spreads out from the singer at ∼1500 m/s (the speed of sound in seawater). When this ring hits sufficiently reflective or resonant targets, distorted and diminished replicas of the unit (echoes) will propagate back to the singer. If a singer hears the echoes, it can potentially use them to determine: (1) that one or more echo-generating targets are present; (2) the distance to detected targets, based on how long it took the echoes to arrive; and (3) the approximate directions to the targets producing the echoes (Schneider et al., 2014). By simulating sound propagation in humpback whale habitats and re-evaluating past behavioral observations of singing humpback whales, Frazer and Mercado (2000) partially confirmed that at least some units in humpback whale songs may generate echoes sufficient for singers to detect and localize whale-sized targets from long distances. Showing that units generate echoes does not prove that singing humpback whales are singing to echolocate. Physics guarantees that most, if not all, sounds produced by singing humpback whales will generate echoes; some echoes will be easy for a singer to detect and others will be undetectable. The novelty of the “song as sonar” hypothesis lies in its proposal that singers are actively listening for song-generated echoes and using them to construct percepts. It is, of course, possible that singers might hear and recognize echoes from units even if this is not why whales sing. However, as evidence accumulates that singers produce sounds in ways that facilitate the generation and reception of biologically useful echoes and perform actions indicative of the detection and use of such echoes, the likelihood that humpback whales sing to actively generate such echoes increases.

The sonar model construes singing in all contexts as an active process of auditory scene analysis in which singers may dynamically adjust unit features and sequences, based on the specific conditions that they encounter, to improve their ability to detect and monitor the actions of conspecifics or other large targets located kilometers away. With regard to the functionality of units, the sonar model offers a significantly different view from sexual advertisement hypotheses. Tyack (1981) argued that the diversity of units produced by singing humpback whales implies that units do not qualify as signals, and that only whole songs are functional (see also Noad et al., 2004). The sonar model, in contrast, considers the acoustic details of units to be fundamental to song function, with variations in the timing, frequency, form, and order of units all serving to facilitate a singer’s ability to construct acoustic images from song-generated echoes. Consequently, the sonar model emphasizes acoustic events that occur during and after the production of each unit within a song, as well as the auditory processes that a singer would need to engage to successfully extract useful information from echoes. The only known way that a humpback whale can detect other whales located more than a kilometer away is by hearing them. Traditionally, researchers have assumed that singers become aware of other whales when those whales either join them or actively make sounds revealing their presence. From the perspective of sexual advertisement hypotheses, songs primarily serve to persuade *potential* listeners to respond to the singer (since in most cases a singer will not know if silent whales are within hearing range or attending to its song). In contrast, the sonar model suggests that singers are not waiting for other whales to reveal themselves but are instead actively searching for conspecifics and attempting to monitor their movements.

### Comparisons With Echolocation by Other Cetaceans and Bats

It is well established that some cetaceans (e.g., bottlenose dolphins, *Tursiops truncatus*) use echoes to identify and locate objects. Nevertheless, many researchers remain skeptical that the largest cetaceans construct percepts from self-generated echoes, other than possibly to detect bathymetric features (Norris, 1966; Tyack, 1997; Tyack and Clark, 2000) or ice sheets (Ellison et al., 1987; George et al., 1988). Instead, most scientists assume that sounds produced by baleen whales are exclusively communicative, and that any resulting echoes will tend to interfere with long-distance communication (Edds-Walton, 1997). Skepticism about humpback whale biosonar partly stems from the fact that humpbacks do not possess adaptations for ultrasonic echolocation and do not produce ultrasonic clicks (Fordyce and Barnes, 1994). Use of ultrasound is not a prerequisite for biosonar, however (see, for example, Brinklov et al., 2013). The sonar model assumes that like other echolocating species, humpback whales evolved biosonar abilities through processes of natural selection driven by sensory constraints.

The use of graded, dynamic sound repertoires for long-distance acoustic communication by animals is quite rare. There is one group of mammals, however, that regularly and continuously modulates the features of their vocalizations along multiple acoustic dimensions as they transmit them over relatively long distances: echolocating bats (Simmons and Stein, 1980; Fenton et al., 2014). The sounds and sequences used by some echolocating bats are strikingly similar to those produced by singing humpback whales (**Figure 1**). Although gradual changes in unit qualities increase signal uncertainty for distant listening whales, these changes can potentially simplify echo processing for the whale producing them, because the singer has direct access to the timing and form of the undistorted units. Furthermore, whereas propagation-related degradation of units confounds between-song comparisons, such distortions within echoes can potentially provide a singer with important information about the locations from which those echoes originated (Tyack, 1997). Frazer and Mercado (2000) proposed that humpback whales might process echoes like bats, by using cortical maps to translate echoes into auditory images of targets (Geva-Sagiv et al., 2015).

The hearing sensitivities of humpback whales have yet to be experimentally measured in any detail, but can be estimated based on similarities between their auditory system and those of other species (Ketten, 1992). In particular, the cochlea and auditory nerve in humpback whales are highly innervated, greatly exceeding the cellular densities of most terrestrial mammals (including songbirds), and matching or exceeding the densities seen in many toothed whales and bats (Ketten, 1997). For instance, the auditory nerve of the humpback whale contains five times more ganglia than are present in humans. The potential resolution of sounds at the earliest stages of auditory processing, which is correlated with neural density, currently provides the best neuroanatomical indication of whether a particular species might possess biosonar. Like other echolocating species, humpback whales possess much higher densities of neurons in their auditory periphery than are typically needed for acoustic communication and passive sound localization.

Bats and dolphins produce and process echoes while actively attempting to intercept and capture small, moving targets. In such search-and-consume contexts, they control the timing of signal production such that echoes from a target arrive during intervals of silence (reviewed by Surlykke et al., 2014). However, when dolphins attempt to detect a stationary target at long distances (Finneran, 2013; Finneran et al., 2014), and when bats are searching for prey (Moss and Surlykke, 2010; Fenton et al., 2012), they produce inter-signal intervals that are more constant and rhythmic. Several bat species use alternating, rhythmically produced sound patterns when searching for prey (Obrist, 1995; Fenton et al., 2014; see also **Figure 1D**), a strategy which may enhance the detectability of echoes within complex environments (Jung et al., 2007; Ratcliffe et al., 2011). Patterned, rhythmic sound production by singing humpback whales may similarly serve to enhance echoic perception (Schneider and Mercado, 2018). Silent intervals between units typically are regularly spaced within song phrases of particular themes (Thompson, 1981, Unpublished). In fact, phrase duration is the most stable feature of songs (Payne et al., 1983). The sonar model assumes that the “silent” intervals between units, which account for ∼50% of song duration, contain acoustic reflections that singers actively process to construct acoustic images of the world around them, and that singers regulate the patterning of sound production to enhance their ability to detect and localize distant targets, like bats and dolphins do.

### How Humpback Whale Singing Differs From Biosonar in Other Species

The kinds of echoic percepts that humpback whales might form using song-generated echoes likely differ significantly from those used by bats and dolphins because of the extensive distances that songs travel underwater, differences in the sounds generating echoes, and the fact that singers are not attempting to capture small prey. Bats are known to use longer duration signals, with energy focused at lower frequencies, when they are engaged in the search phase of echolocation (Simmons and Stein, 1980). The sonar model assumes that humpback whales are searching for much larger targets from much longer distances than bats, and that they integrate echoic information over much longer periods. Many of the ways that singing differs from bat (and dolphin) biosonar can be linked to the novel challenges that singers face in processing low-frequency echoes from large targets that arrive from multiple directions after propagating several kilometers in shallow water environments, and that can potentially be masked by the songs of other whales.

#### Environmental Conditions

The situation faced by a humpback whale attempting to interpret echoes while singing corresponds to a version of the “cocktail party problem,” similar to the one faced by bats that echolocate within swarms, in which one or more echo streams must be isolated from a mixture of many simultaneously received sound streams with similar acoustic features (Warnecke et al., 2015). Humpback whales may have evolved ways of solving this problem that differ slightly from those used by either bats or dolphins. For instance, singing humpback whales sometimes adopt a stereotyped position in the water column when singing that likely affects how their sounds propagate (Au et al., 2006; Sousa-Lima, 2007), as well as which echoes they are most likely to detect (Mercado and Frazer, 1999). Singers may also seek out environments with particular features (e.g., positioning themselves near banks) that affect how their units propagate (Winn and Winn, 1978; MacKay et al., 2016).

#### Search Space

**FIGURE 3:**
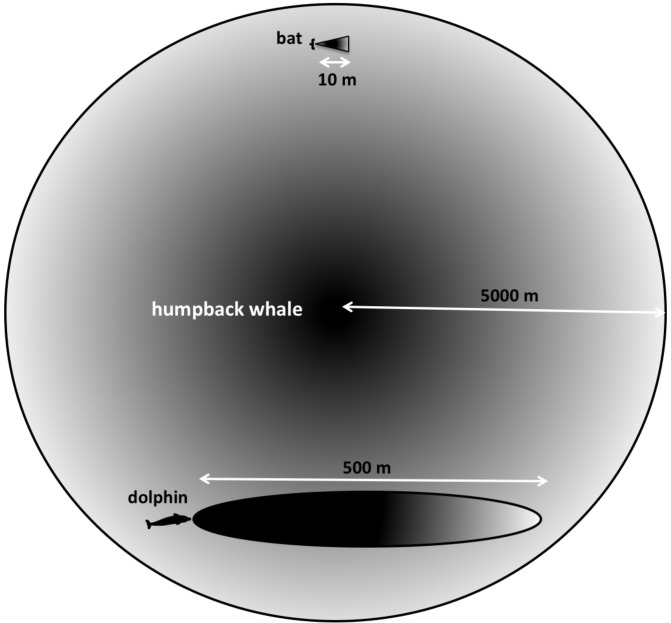
Searching at different scales. “Long-range” for a bat searching for centimeter-sized targets in air is approximately 10 m, whereas dolphins can detect multi-centimeter-sized targets located hundreds of meters away. Because both bats and dolphins use ultrasonic signals, their sonar beams are highly directional. Humpback whales’ lower frequency signals are more omnidirectional, and potentially can enable them to detect multi-meter-sized targets at much farther distances.

Humpback whales interact over distances of at least 9 km (Tyack, 1981). The longest ranges that bats are known to echolocate over are less than 10–20 m (Simmons et al., 2014). Bottlenose dolphins are thought to have a maximum range on the order of 800 m (Finneran, 2013). Many targets of interest to a singing humpback whale may be located thousands of meters away (**Figure 3**). Additionally, the sounds produced by singing whales are much less directional than the sonar signals used by either bats or dolphins. Consequently, singing humpback whales likely experience echoes returning from multiple directions simultaneously.

#### Time

The short-duration sonar signals used by bats (∼1–50 ms) and toothed whales (∼10–200 μs) increase the precision with which echo arrivals can be timed, as well as the rate at which discrete echoes can be received (Fenton et al., 2014). Units produced by singing humpback whales are much longer (∼1–3 s), making them suitable for transmission over quite long distances, but with significantly reduced temporal resolution. Consequently, humpback whales are unlikely to use echo delays to estimate target distances as precisely as bats or dolphins do. If singers time their unit production such that relevant echoes arrive within intervals between units, then one might expect interval durations to correlate with target distance. When bats are in the search phase of echolocation, however, their inter-signal intervals are more likely to indicate that they are scanning at their maximum search range (Moss et al., 2014). If singing humpback whales are actively searching, then their inter-unit intervals may correspond to the radius of their search space; intervals between units with matching acoustic features can be 10 s or longer, suggesting maximum detection ranges of 7 km or more (because a unit travels ∼15 km in 10 s). Singers rhythmically produce units in much longer bouts than echolocating bats or dolphins, indicating that they may either detect targets at lower rates or require more time to extract relevant information from echo streams.

#### Signal Form

Bats and dolphins make use of a fixed set of sonar signals with specific acoustic properties that have been highly refined through evolutionary processes to yield predictable echoic features (Simmons and Stein, 1980). Singing humpback whales, in contrast, seem to use a more varied repertoire of units, which they produce in an exceptionally wide range of soundscapes; the acoustic properties of units within a song can vary from highly stable to highly dynamic (Mercado et al., 2010; Mercado, 2016). Use of ultrasound by bats and dolphins enables them to rapidly extract fine-resolution spatial details from echoes. These acoustic properties are less suitable for searching a large volume of the ocean, which is why military sonar systems typically make use of lower frequency sounds when attempting to detect submarines at long distances (Decarpigny et al., 1991; Denny, 2007). The specific acoustic signals that would be suitable for use as echolocation by humpback whales searching over long distances may differ significantly from the sounds used by echolocating bats and dolphins. Alternatively, if one normalizes for differences in the search distances and target sizes, then many of the signal features that facilitate echoic perception by bats may also be advantageous for singing humpback whales.

### Are Humpback Whale Songs Generating Echoic Percepts?

Critics of the sonar model have argued that it is impossible for humpback whales to echolocate using songs because: (1) the sounds within songs are not suitable for use as sonar—they are too variable and not sufficiently intense; (2) the complexity of song structure is inconsistent with a sonar function; (3) the constantly changing nature of humpback whale songs precludes the possibility that they could be used for sonar; (4) any echoes from other whales would be masked by ambient noises, other songs, and reverberation; (5) song-generated echoes cannot be discriminated from song sounds produced by distant whales; (6) the complexity and variety of environments within which humpback whales sing confounds reliable recognition of echoes; and (7) humpback whale social behaviors are more indicative of song being a breeding display (Au et al., 2001). Others have questioned why, if humpback whale songs are effective as sonar signals, females don’t ever sing. Also, why don’t humpback whales sing all the time, especially when they are migrating and need to navigate over long distances? And finally, why are singers constantly converging on a shared song form even as they progressively change song structure, and how could this possibly help them to construct percepts from echoes?

Comparable critiques and questions were raised when researchers first suggested that bats might echolocate using inaudible sounds (Griffin, 1958), an hypothesis that initially seemed inconsistent with well-known facts about sound and auditory perception. Earlier papers have addressed most of the concerns noted above, at least in part (Frazer and Mercado, 2000; Mercado and Frazer, 2001), and so they will only be briefly discussed here. Most past assessments of song complexity, including those properties that change over time, are based on subjective classifications of units and phrases that do not account for the features of units that are functionally relevant if songs are used for biosonar. The soundscapes within which humpback whales sing clearly can affect the detectability of echoes. All echolocating animals face the problem of extracting self-generated echoes from complex acoustic scenes. Rather than assuming *a priori* that this problem is insurmountable for humpback whales, the sonar model assumes that the acoustic properties of song, combined with sophisticated auditory processing mechanisms, may make this problem solvable. How units may generate useful percepts and how song structure may facilitate echo reception will be considered in later sections.

Singers on the breeding grounds are much more likely to be alone than to be socially interacting with other whales (Winn and Winn, 1978; Darling et al., 2006; Herman, 2017), and the most commonly observed social interaction associated with singing (a single *male* briefly joining a singer) is rarely observed in animals performing breeding displays. Observations that humpback whales sometimes sing while accompanying females with calves have led researchers to question whether songs play any role in searching (Au et al., 2001). However, this critique assumes that males find all females equally attractive, regardless of their sexual receptivity, and that singers are uninterested in the movements of any other whales once they encounter any female. The sonar model assumes that singing is an active perceptual process, and thus only weakly constrains the range of social situations within which a whale might sing. Nevertheless, the more varied the behavioral contexts within which humpback whales sing, the more parsimonious the sonar model becomes relative to the hypothesis that songs are acoustic displays that serve multiple reproductive functions.

The fact that a humpback whale can potentially gain useful perceptual information from song-generated echoes does not imply that they can only obtain echoic information by singing. In principle, a whale might also gain useful information from producing simpler sequences, repeating a single sound, or producing irregular sequences. The sonar model assumes that singing is a persistent mode of sonar signal production that is particularly well suited for detecting and tracking movements of large, distant targets (analogous to an air traffic control radar system). To the extent that female humpback whales or other species of baleen whales gain advantages from perceptually monitoring targets over large areas, the model specifically predicts that they should “sing.” A possible explanation for the fact that female humpback whales are rarely observed singing in breeding contexts is that they are generally less motivated to join conspecifics than males are. Whales singing on feeding grounds have not been sexed, so it remains possible that many are females. It was once thought that only male songbirds sang, but it is now known that both males and females sing in most species (Odom et al., 2014). No echolocating species uses sonar constantly and it is not clear that the modes of biosonar that work well for tracking multiple whales at long distances would necessarily be advantageous for navigation during migration.

The most puzzling aspect of humpback whale songs from the perspective of the sonar model relates to the progressive changes that singers make over time. Unlike sexual advertisement hypotheses, the sonar model assumes that such changes cannot be arbitrary if units and phrases are to remain functional. The model proposes that such changes occur as singers seek to avoid mutual interference (discussed below), but it does not predict how singers will converge or diverge while singing or explain specifically how this might facilitate echo processing.

## New Evidence Supporting the Sonar Model and Weakening Sexual Advertisement Hypotheses

In the 20 years since the sonar model of humpback whale song was first proposed, additional evidence has accumulated that confirms several assumptions and predictions of the model, highlights further limitations of sexual advertisement hypotheses, and clarifies how humpback whales may use biosonar. Acoustic observations, in particular, are beginning to reveal the full potential of humpback whale songs as echo-generating signals.

### Humpback Whales Act Like Other Echolocating Animals

Behavioral studies of singing humpback whales have focused on identifying how singers interact with other whales (Herman, 2017). The sonar model is more concerned with clarifying what singers perceive than with explaining why they choose to engage in certain actions in different situations. Many factors will determine how a singer reacts to information gained echoically, just as various factors will affect a singer’s response to visual information. Consequently, the model does not dictate how individual singers will behave while singing. The main behavioral predictions of the sonar model are that humpback whales will be most likely to sing in situations where monitoring the movements of silent, distant whales would be advantageous, and that in some cases they will use this information to intercept detected whales. Darling and Berube (2001) and Darling et al. (2006) confirmed that males near Maui sing for hours while alone and semi-stationary, and that they occasionally stop singing before swimming to join other distant whales. Whales swimming in the vicinity of singers were found to “lower their voices” (Dunlop, 2016), presumably to reduce the likelihood that singers would hear and localize them, further highlighting the challenges singers face in detecting and joining distant whales. When other whales approach singers, the joiners are usually lone males (Darling et al., 2012), in direct contradiction to what most sexual advertisement hypotheses predict. When a silent male joins a singer, the singer typically stops singing, interacts non-aggressively with the male for 5 min or less, after which the two males usually separate. Such interactions are atypical of males that use acoustic displays as sexual advertisements, leading to the novel proposal that songs might serve as communicative signals that facilitate cooperative affiliations between males (Darling et al., 2006). Evidence of males joining singers does not directly support the sonar model but does provide grounds for rejecting or radically revising current sexual advertisement hypotheses. Although the sonar model does not address how conspecifics should respond to a singer, in other echolocating species conspecifics often approach and join other individuals by eavesdropping on their sonar signals (Balcombe and Fenton, 1988; Fenton, 2003).

The sonar model specifically predicts that humpback whales will sing when they need to monitor multiple whales that they cannot see, and thus predicts that both males and females may sing in certain feeding contexts (e.g., to avoid collisions). Recordings from tagged humpback whales feeding in Antarctica revealed that they were singing while simultaneously engaging in deep dives (100+ m) that culminated in feeding lunges (Stimpert et al., 2012). Other evidence suggests that humpbacks may “sing” to detect prey. The movements of a single fish are less relevant to a humpback than are the movements of fish schools or other whales. Recent studies show that humpback vocalization levels are correlated in space and time with densities of herring (Gong et al., 2014; Huang et al., 2016; Wang et al., 2016), and that the sounds humpback whales produce during high fish densities are adequate for detecting fish schools over kilometer distances (Yi and Makris, 2016). Interestingly, some tonal sounds that humpback whales produce when foraging on herring are of similar duration, frequency content, and intensity as song units, and are repeated at regular intervals (∼14 s), meeting the criteria that biologists use to classify vocalizations as songs (Spector, 1994). Finally, humpback whales are now known to produce click trains while foraging at night (Stimpert et al., 2007), varying their inter-click interval such that the shortest intervals occur just before they perform feeding lunges, just as echolocating bats and dolphins do when they intercept prey. These findings suggest that humpback whales, like other echolocating animals, are able to flexibly adjust the timing and form of their sounds as needed to match the conditions within which they are vocalizing, and that they commonly sing in contexts, seasons, and locations that are not traditionally associated with breeding.

### Units Are Well Suited for Long-Range Sonar

The detectability of song-generated echoes is constrained by the intensity of units within songs. In initial evaluations of the sonar model, we estimated that units might reach source levels of 185 dB re 1 μPa at 1 m (Frazer and Mercado, 2000). Subsequent detailed acoustic measurements of three singers revealed that their loudest units reached source levels of 184 ± 4 dB on average, confirming our initial estimates (Au et al., 2006). Units recorded from larger numbers of whales singing on feeding grounds were 155–205 dB (Gong et al., 2014). These levels are sufficient to generate significant echoes at kilometer distances. Toothed whales use more intense sonar signals (220–240 dB) to detect targets over shorter distances (Wahlberg and Surlykke, 2014), which might seem to suggest that units are too quiet to generate useful echoes. However, energy within toothed whale sonar signals is spread across a broad band of ultrasonic frequencies within a brief impulsive sound, whereas the energy within the tonal units produced by singers is concentrated in long-lasting, narrow-frequency bands, like the sonar signals bats use when searching for larger targets at longer ranges (Simmons and Stein, 1980).

**FIGURE 4:**
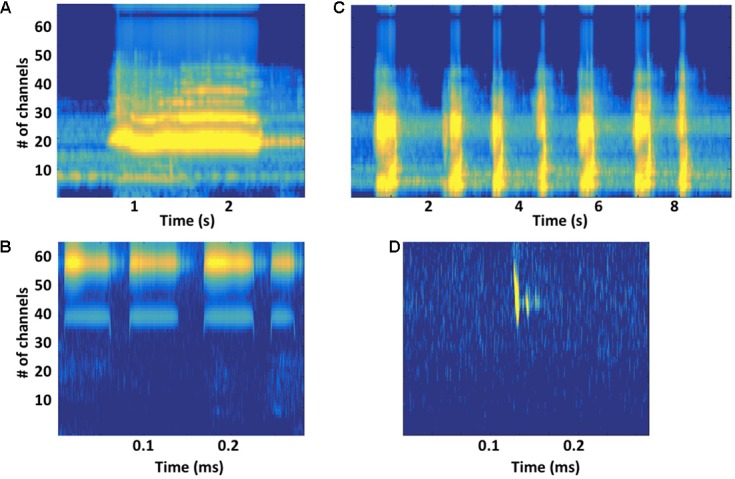
**(A)** Gammatone-based spectrographic image (gammatonegram; Ellis, 2009) of a constant-frequency (CF) unit produced by a singing humpback whale, with gammatone filter characteristics matched to an estimated humpback whale cochlear frequency position function (Branstetter and Mercado, 2006; Branstetter et al., 2007), approximates unit registration at a humpback whale’s ear. **(B)** Gammatonegram of a packet of broadband units produced by a singing humpback. **(C)** Gammatonegram of bat (*Rhinolophus mehelyi*) CF sonar signals, with filter characteristics matched to an estimated bat cochlear frequency position function (Muller and Schnitzler, 2000). **(D)** Gammatonegram of a bat (*Pipistrellus pipistrellus*) frequency-modulated sonar signal (followed by two echoes). Note that time scales are much smaller for bat sonar signals than for humpback whale song units.

Acoustic features of units cannot provide compelling evidence that songs function as sonar signals, but their properties do place constraints on the kinds of echoes singers can potentially generate (Frazer and Mercado, 2000). Large variations in unit features suggest that different unit types may play different functional roles (Winn and Winn, 1978). Units scaled using estimated humpback whale cochlear sensitivities (Branstetter and Mercado, 2006; Mercado et al., 2008), show constant-frequency (CF) and frequency-modulated (FM) features comparable to those present in the sonar signals of bats (**Figure 4**). These shared acoustic properties further suggest that singing humpback whales may produce echo streams similar to those produced by echolocating bats. Detailed acoustic analyses of song units have confirmed that singers are not using a fixed repertoire of discrete sound types (Mercado et al., 2010; Cazau et al., 2013), but are instead continuously varying units along multiple acoustic dimensions (**Figure 1A**). Use of a graded, continuously varying sound repertoire by singers is inconsistent with the proposal that females (or males) assess male fitness by remotely comparing songs, because females have no way of determining which variations in received songs are the result of propagation-related distortion. Graded shifts in unit features (particularly FM and frequency content) can enhance propagation in different environmental conditions and at different ranges, which is why echolocating bats often morph their sonar signals (Fenton et al., 2014).

### Echoes From Units Are Long-Lasting

**FIGURE 5:**
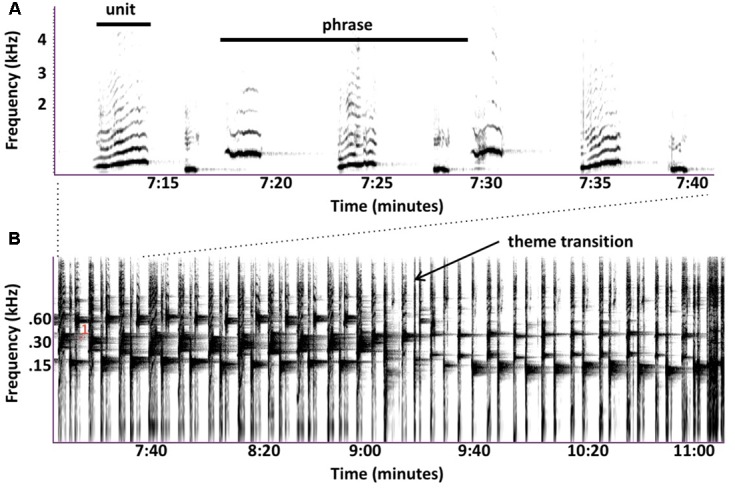
Spectrograms of humpback whale unit sequences, recorded in Maui by D. Rothenberg in 2007 (Rothenberg, 2015). **(A)** Three-unit phrases shown as traditionally depicted and analyzed (Fast Fourier Transform = 1024, 50% overlap, linear frequency axis). **(B)** ∼4 min sequence including these same phrases (Fast Fourier Transform = 10240, 50% overlap, log frequency axis) highlighting persistent narrowband reverberation from units. Vertical bands correspond to units and horizontal bands are unit-generated reverberation. Temporal patterning is evident from the regular spacing of units and spectral interleaving is shown by the spacing between reverberant bands from each of the three types of units. Note that when the singer transitions between themes, he gradually morphs rhythmic and spectral phrase features, maintaining the pace of sound production as well as elements of spectral patterning.

Singing humpback whales produce large numbers of units with CF or quasi-CF (minimally FM) components (Ou et al., 2013). In at least some environments, these units can generate spectrally narrow, reverberant bands (overlapping environmental echoes) that persist 10 s or more, such that acoustic energy within the band lasts for as long as the singer repeats a phrase (Mercado, 2016). Units immediately following CF units often contain acoustic energy focused at frequencies just above or just below the CF band (**Figure 5**). In other words, singers spectrally separate consecutive units within phrases, as is seen in echolocating bats (Jung et al., 2007; Ratcliffe et al., 2011), thereby decreasing “cross-contamination” between echoes generated by those units.

Reverberant bands are superposed echoes reflecting from multiple directions and distances. Traditionally, reverberation has been viewed as a source of interference that can potentially degrade communication and echo detection. However, some species of bats actively generate narrowband reverberation when echolocating (Henson, 1987; Fenton et al., 2012). These bats (called high duty cycle bats) repetitiously produce CF signals with short inter-pulse intervals such that they constantly receive echoes from those signals, even while signals are being produced. This mode of echolocation is prevalent when bats are attempting to detect weak echoes. By listening for changes in the frequency and amplitude of a continuous stream of background echoes, a perched bat can detect prey as it flies through the bat’s search space. Whales entering the search space of a singer might similarly lead to changes in song-generated reverberant bands (LePage, 1998).

Repetitious production of long-duration, CF units by singers is consistent with the sonar model because such signals should facilitate the detection of weak echoes in complex acoustic backgrounds. Spectral interleaving of units is also consistent with the model, because this can potentially extend the range of echo detection (Jung et al., 2007). The recent discovery that some CF units reliably generate narrow reverberant bands within frequency ranges that are near-optimal for long-distance shallow water propagation, along with the finding that singers spectrally interleave units, provides the strongest acoustic evidence to date that some units function primarily to generate echoes.

### Progressive Changes in Songs Are Predictable

Au et al. (2001, p. 298) argued that since song structure “changes completely within about 5 years, such that songs recorded in the same location about 5 years apart will not have any song units in common,” that the sonar model implies that singers use a “continuously inefficient sonar system.” If singers change song structure arbitrarily over time, then this critique is valid and the echo-generating potential of songs should fluctuate across years. The sonar model thus predicts that singers will not arbitrarily modify the acoustics of songs over time but will only make changes that maintain the utility of song-generated echoes. Accumulating evidence from acoustic analyses suggests that although song changes can be extensive, they are not arbitrary. In particular, singers progressively modify the acoustic qualities of some units across entire songs (Mercado and Sturdy, 2017). For example, the frequency content of units often gradually shifts to lower frequencies as a song cycle progresses (Mercado et al., 2010; Mercado and Handel, 2012; Kowarski et al., 2018). Although gradual shifts in phrase structure are evident within and across years (Payne et al., 1983; Payne and Payne, 1985), the acoustic relationships between consecutive units appear to be stable across years and populations (Green et al., 2011; Mercado, 2016). In fact, objective analyses of phrase structure show several commonalities (e.g., prototypical modes of unit alternation) across years and populations (Mercado et al., 2003; Mercado, 2016). In short, sequencing of both units and themes within songs is more consistent over time and locales than past literature suggests. These findings show that singing humpback whales are acoustically constrained in how they progressively change song structure over time, as predicted by the sonar model.

Singing humpback whales adjust song content based on songs they hear other whales producing (Cholewiak et al., 2018), and based on non-song sounds that they experience while singing (Miller et al., 2000; Fristrup et al., 2003). The sonar model explains all such changes as modifications that singers make to reduce interference with echo processing. The model does not explain why singers progressively morph their songs over months and years, a strategy that no other echolocating animals use. Proponents of sexual advertisement hypotheses suggest that songs progressively change because: singers copy any new song variants that they hear (Noad et al., 2000). The assumptions underlying this interpretation are that some singers are more innovative than others and that innovative songs attract more females (Payne, 2000; Parsons et al., 2008; Janik, 2009). However, detailed analyses of song changes contradict the claim that changes arise from singers copying innovators. For instance, whales singing off the coast of Mexico progressively changed acoustic features of their songs in parallel with singers in Hawaiian waters, without being exposed to those singers (Cerchio et al., 2001; see also Noad et al., 2004). The introduction of innovative features by individual singers should lead to greater divergence in song structure between acoustically isolated whales. Parallel changes in song structure across acoustically separated groups suggest that singers progressively modify songs in ways that are either deterministic or innate. Additionally, studies of singing in the South Pacific revealed that song changes spread unidirectionally, with whales in French Polynesia producing songs that whales in East Australia sang in the previous year (Garland et al., 2011). Such geographically directional cultural transmission would imply that only the most westerly singers are innovators, which seems unlikely.

## Evaluating and Revising the Model

Modeling humpback whale biosonar as a process of detecting and interpreting individual echoes has the advantage of simplicity but is limited in several respects. First, the original model does not directly address how variations in the qualities of units might affect the kinds of information that echoes provide. Second, the model does not account for the fact that different sound channels will distort echoes in different ways, complicating echoic perception. Finally, the model does not explain how regularities in the timing and sequencing of different units might contribute to echoic perception. Here, a modified version of the sonar model is proposed to better capture these aspects of humpback whale singing. The revised model proposes that singers generate multiple concurrent echo streams, some specialized for target detection and others for target localization and classification. The revised model also makes several novel, testable predictions (described below) that can be explored in future studies to objectively evaluate its value and validity.

### Signal Specializations for Detection Versus Localization

Bats echolocate using two main classes of sonar signals: short duration, broadband FM sounds—typically used at shorter ranges—and narrowband, long duration CF sounds, which are prevalent during searching (Fenton et al., 2014). Processing of echoes from these two signal types has been described as involving “two distinct kinds of acoustic imaging systems” (Simmons, 1989). CF signals are well suited for long-range detection of echoes, but make it difficult to pinpoint echo arrival times, decreasing range resolution. Conversely, FM signals provide more details about target distances and target features, but at the cost of decreased long-range echo detectability. Humpback whale song units also contain CF and FM elements, as well as less stereotyped features including complex FM sounds, frequency jumps, pulse trains, and chaotic noise-like bursts (Mercado et al., 2010; Cazau et al., 2016). Units with CF or quasi-CF (qCF) components are ubiquitous within humpback whale songs. Acoustic energy within CF/qCF units is typically concentrated within one or two narrow frequency bands (Mercado, 2016). Packets of multiple, short-duration (<0.5 s), broadband/FM sounds with comparably short inter-unit intervals are also common (Mercado et al., 2003).

Broadband/FM units appear to vary more than CF/qCF units in their form, timing, and number of repetitions within song sessions (Winn and Winn, 1978). Variations in broadband unit features may serve to counteract destructive interference that occurs during underwater propagation, control the directionality of acoustic fields, or enhance the detectability of echoes from specific ranges. The revised sonar model retains the assumption of the original model that singers identify echoes from broadband/FM units by matching them to recently produced units, and that echo delays and spectrotemporal distortions enable singers to estimate target positions from such echoes.

Singers repeat some CF/qCF units at regular intervals that are longer (∼7–16 s) than the typical intervals between broadband/FM units (Mercado, 2016). Long-lasting reverberant bands generated by such units can persist throughout a phrase such that energy within a narrow band may persist for 20 min or more. Production of continuous narrowband reverberation by singing humpback whales is reminiscent of echolocation by high-duty-cycle CF bats (Henson, 1987). CF bats have exceptional frequency resolution capacities that enable them to detect small changes in background echoes caused by targets. The revised sonar model proposes that singing humpback whales monitor continuous, long-lasting echo streams from CF/qCF units separately from, but in parallel with, processing more temporally discrete echoes generated by broadband/FM units, thereby maximizing both the detectability and localizability of targets.

This “duplex” sonar model (**Figure 2B**) makes several novel predictions about the repertoire of units that singers should use. First, it predicts that distributions of inter-onset intervals for acoustically similar CF/qCF units will differ systematically from those of broadband/FM units. Second, the model predicts that unit bandwidth will be negatively correlated with unit duration, as it is in bat biosonar^1^. Third, it predicts that CF/qCF sounds comparable to those produced by singers can be used to detect whale-sized targets at longer distances than unit-like broadband/FM sounds, but that the latter will generate more localizable echoes. Finally, the duplex model predicts that the proportion of CF to broadband units within songs will be context-dependent. When few or no relevant targets are within detection range, CF units should be more prevalent. When targets are being actively monitored, the proportion of broadband units should increase. These untested predictions, which are unique to the duplex sonar model, provide a way to test its accuracy.

### Recognizing Echoes Versus Detecting Novelty

Many past biosonar models have focused on understanding how animals interpret echoes from individual sounds. Tracking targets, however, requires integrating information from multiple echoes (Moss and Surlykke, 2010). Integrating changes in echoes over time would be especially critical for a humpback whale attempting to monitor distant targets. For example, a singer that detects another whale swimming 5 km away at 4 km/h would need to predict that whale’s location ∼20 min into the future to intercept it. Field observations show that lone humpback whales often sing continuously for hours at a time, with the longest documented song session lasting 22+ h (Winn and Winn, 1978). A whale singing 20 h produces ∼20,000 units and as many silent intervals. Because lone singers are often stationary or slow moving (Frankel et al., 1995; Henderson et al., 2018), the acoustic background of echo streams generated by songs at a particular location should become highly familiar to a singer, which may in turn facilitate the detection of targets that enter a singer’s search space.

The benefits of familiarity with a perceptual background have been established both for visual search (Reicher, 1976; Richards and Reicher, 1978; Mruczek and Sheinberg, 2005), and for the detection of auditory targets (Feng and Oxenham, 2015). Numerous studies have also shown that mammalian auditory systems are highly sensitive to mismatches within repetitious sound sequences (Escera et al., 1998; Naatanen et al., 2007), and that mammals commonly orient to novel sounds (Van Olst, 1971). Familiarity can potentially enhance target detection in any environment. Introduction of a novel source of echoes into a familiar soundscape may lead to perceptual pop-out (Wang et al., 1994). Importantly, a singer can potentially gain useful information from echoes generated by whales entering its search space, *even if the singer does not recognize the echoes*. Once a singer detects an echoic anomaly, continued monitoring of relative changes in that anomaly could reveal a target’s movements.

Detecting novel echoes within a familiar auditory scene requires different auditory computations from those involved in recognizing distorted signal replicas or calculating echo delays. This type of echolocation has been described as interferometry because patterns of interference caused by superposed acoustic waves lead to changes in auditory percepts (Henson, 1987). For instance, CF bats detect “acoustic glints” of interference caused by Doppler-shifted echoes generated by the fluttering wings of insects, as well as periodic amplitude modulation caused by outgoing pulses (or other echoes) overlapping with incoming echoes. Resonance within a bat’s ear can contribute to such interference patterns, as can large environmental reflectors. CF bats are able to detect and classify subtle frequency and amplitude modulations in echo streams, in part, because of their densely innervated inner ear. Notably, the density of neurons per mm along a humpback whale’s basilar membrane (∼2,400 cells/mm) is higher than the highest densities (∼1,900 cells/mm) in the ears of CF bats (Ketten, 1997), suggesting that humpback whales also possess the cochlear resolution necessary for detecting subtle patterns caused by interference from echoes. If humpback whales are not able to echolocate, then they are an evolutionary anomaly because no non-echolocating vertebrate has evolved such high-resolution auditory reception. The duplex sonar model suggests that this adaption serves specifically to enable singers to make fine distinctions between the patterns of song-generated reverberation that are present when no target is within the singer’s search space versus when one or more targets are moving through that space.

The kinds of acoustic interference patterns that might be informative to a humpback whale are likely to differ from those that are salient for CF bats. But, the strategy of searching for deviations within continuous streams of echoes (e.g., song-generated reverberation) may still be effective. If singing humpback whales produce CF/qCF units to generate interference patterns that facilitate the detection of large targets at long ranges, as proposed in the duplex sonar model, then one would expect: (1) to find anatomical specializations in the auditory periphery of humpback whales that enhance the reception and detection of such patterns; (2) that stationary singers would tend to position themselves at locations that are conducive to reverberation and/or that produce strong background echoes; (3) that physiological recordings of cochlear potentials in singing humpback whales would reveal strong responses to interference patterns associated with whales swimming through a singer’s search space; and (4) that highly reverberant, long-duration, CF unit sequences provide an adequate source for detecting the presence of non-singing whales in humpback whale habitats when modulations in reverberation from those signals are continuously analyzed using a system designed to detect deviations in interference patterns. Testing these predictions in future studies can further clarify the adequacy of the duplex sonar model.

### Jamming Avoidance

Singing humpback whales can partly control the form and timing of song-generated echoes. The detectability and discriminability of echoes also depends on the actions of other singers, however. Specifically, songs produced by other whales may interfere with echoic perception. In certain months and locales (e.g., off the coast of Maui in winter), chorusing by multiple singing humpback whales can be heard (Au et al., 2000). Choruses are cacophonous with no apparent coordination between singers, a formidable signal-processing problem for any singer attempting to monitor its own song-generated echo streams. This auditory challenge is simpler in certain respects, however, than the one faced by any non-singing whales attempting to assess the fitness of singers within such choruses. A singer knows exactly what units and unit patterns it is producing, and can flexibly adjust the timing, duration, and spectral content of units while listening for matches or deviations within relatively short time windows. If there is any ambiguity about whether a received sound is an echo or another singing whale, the singer can adjust its units to see if the echoes change accordingly. In contrast, a whale attempting to compare songs produced within a chorus must parse and spatially separate multiple overlapping sequences, assign them to specific singers, keep track of all the differences in the sequences of each singer while discounting any variations caused by propagation-related distortion, and compare these differences across long periods. Recent comparative studies have revealed a variety of mechanisms that animals use to perceptually organize complex auditory scenes (Hulse, 2002; Bee and Micheyl, 2008; Lewicki et al., 2014), and to avoid mutual interference (Fawcett and Ratcliffe, 2015; Warnecke et al., 2015; Adams et al., 2017), several of which may be used by humpback whales to either sort and track echoes or to segregate multiple songs within choruses.

If singers in choruses simultaneously produce spectrally matching CF units, then incoming units from other whales could potentially interfere with echo processing (called jamming). Similar issues are encountered by swarms of echolocating bats (Simmons et al., 2004; Moss and Surlykke, 2010), indicating that at least some animals have solved this problem. One strategy that bats use to avoid jamming is frequency switching, either by hopping from one frequency to another and then restricting attention to echoes at the current frequency (Moss and Surlykke, 2010), or by morphing the frequency characteristics of outgoing signals to make them more distinctive (Hase et al., 2018). No non-echolocating vertebrate is known to adjust their signals in this way in response to conspecific vocalizations. On the contrary, birds and terrestrial mammals that use sounds as sexual advertisements typically call in ways that preclude any overlap (taking turns), maximize overlap (through synchronization), or that actively jams a competitor’s signal (Tobias and Seddon, 2009; Naguib and Riebel, 2014). The duplex sonar model thus makes the unique (untested) prediction that singing whales with overlapping search spaces will avoid simultaneously using CF units centered at the same frequency. The model also makes the novel prediction that if tonal sounds centered at a singer’s CF frequency are broadcast within its search space, then the singer will either modify the focal frequency of its CF units or stop singing. Finally, the duplex sonar model explains spectral interleaving of units as avoidance of self-interference, thus predicting that externally provoked shifts in a singer’s CF unit frequency will be accompanied by similar shifts in the frequency content of surrounding broadband units.

A second strategy that some bats use to separate echo streams is to produce their sonar signals in regularly timed patterns (Obrist, 1995). Temporal patterning of sounds can enhance perceptual grouping of similar sounds and facilitate asynchrony across individuals. Signal processing approaches to separating overlapping humpback whale songs also benefit from spectrotemporal patterning of units (Zhang and White, 2017). The original sonar model assumed that regularly timed production of units is critical to echo recognition. The revised model predicts that inter-onset intervals for broadband units, in particular, will be shorter and more uniformly spaced in songs produced within choruses. Cyclical progression through phrase variants might also become more uniform within choruses since this should make it easier for singers to desynchronize their individual song cycles. Recent analyses of song recordings in which multiple whales can be heard singing provide some evidence that singers modify how they cycle through phrases based on what they hear nearby singers doing (Cholewiak et al., 2018). Real-time modulation of progression through phrases may enable singing humpback whales to proactively avoid mutual interference (Parsons et al., 2008).

## Conclusion

Winn and Winn (1978, p. 113) concluded their seminal report on humpback whale songs by noting that, “many of the design features appear to be related to the whales’ constant need for information.” Proponents of sexual advertisement hypotheses suggest that song qualities serve primarily to inform other whales (especially females) of a singer’s fitness. The sonar model instead proposes that songs function primarily to inform singers of what is happening beyond their visual range. Both viewpoints are at least of heuristic value. The sonar model is compatible with the possibility that songs play multiple communicative roles, including informing other whales of a singer’s location and interest in mating. Where the model differs from sexual advertisement hypotheses is in its assumption that humpback whales sing primarily to provide *themselves* with information. Unlike advertisement hypotheses, which often assume that each different context within which humpback whales sing is evidence of a different reproductive function (Herman, 2017), the sonar model assumes that singing is driven by a common motivation in all contexts—an intrinsic motivation to generate percepts that can guide future actions. In the case of males singing in tropical regions where receptive females may be present, the males presumably sing to increase their opportunities for mating (as assumed by sexual advertisement hypotheses). Given that males cannot easily predict when or where a receptive female humpback whale will be, it is likely that they are often searching for such females. Some sexual advertisement hypotheses assume that songs function as search signals, by either attracting females or enticing them to make sounds that reveal their positions (Herman, 2017). Others assume that males alternate between singing and searching, because vocalizing can interfere with the detection of sounds produced by females, and because males on the move are less likely to sing. The sonar model assumes that humpback whales sing to perceptually scan the waters around them.

Studies motivated by the original sonar model have clarified mechanisms of humpback whale sound production and perception, generated testable predictions, led to a deeper appreciation of how units propagate in humpback whale habitats, and promoted critical discussion of the roles that songs play. The revised sonar model builds on recent discoveries, expanding the range and specificity of predictions and providing a novel framework for further study of humpback whale bioacoustics. The duplex sonar model seeks to explain mechanistically why singing humpback whales produce patterned sound sequences by characterizing the physical consequences of singing, clarifying and evaluating the auditory signal processing problems that singers face, and comparing their vocal behavior and auditory capacities with those of other vocally flexible mammals who operate in visually constrained environments.

From the perspective of the sonar model, humpback whale singing is more comparable to bat biosonar than to singing by birds. When echolocating bats search for distant targets within complex acoustic environments, they sometimes use stably timed patterns of spectrally interleaved sounds (e.g., Mora et al., 2011), suggesting that patterned signals provide some advantages over simpler sequences when it comes to echolocating in complex conditions or at long distances. Like humpback whales, some bats remain stationary and alone while producing long series of regularly paced tonal sounds, with energy tightly focused within a narrow frequency band, that generate continuous echo streams. Bats also flexibly adjust the frequency, timing, frequency modulation, and intensity of their sounds depending on the context, and thus, like humpback whales, use a graded repertoire of sounds. One thing that may differentiate singing humpback whales from echolocating bats is that singing whales appear to be predominately males. This may be true during breeding periods, but it remains unclear whether singing is sexually dimorphic in non-breeding contexts. Given that there are no known species in which only males echolocate, the sonar model predicts that female humpback whales will be observed singing in contexts where they can benefit from localizing distant targets and in which there is no cost to them for broadcasting their location.

From an ecological perspective, convergent evolution of perceptual and neural capacities across cetaceans and bats is unsurprising. It is also not surprising that different species of cetaceans might evolve different modes of echolocating, especially given the variety of signals and strategies evident in bats. Initial comparisons of humpback whale songs to birdsongs were motivated, in part, by the complexity of whale songs and their prevalence on the breeding grounds. Humpback whale singing is less seasonal than was originally assumed, however, and whale songs differ acoustically from bird songs in non-trivial ways. Traditionally, the mating system of humpback whales has been considered to be quite complex and somewhat aberrant in that no terrestrial mammals, and few aquatic animals, have evolved comparable acoustic displays. If songs are not breeding displays, however, then the mating strategies of male humpback whales are remarkably similar to those of their close relatives: mountain sheep (Geist, 1971; Darling, 1983) and ibex (Willisch and Neuhaus, 2009).

Humpback whale songs are famous for their complexity but hidden within that complexity are stable properties characteristic of most songs. Payne and Payne (1985) described such properties as rules of song form, including: (1) cycles of ordered themes typically last 8–16 min; (2) songs always contain units that vary along multiple acoustic dimensions; (3) theme cycles may be repeated many times without interruptions in song sessions; (4) the number of phrase repetitions often varies across repeated themes; and (5) some phrases are repeated with minimal modifications and others gradually morph with repetition. Universal features of songs provide important clues about what singers are doing, and about the kinds of echo streams that songs can generate. According to the sonar model, universal features of songs (especially temporal regularity) enhance the segregation, classification, and localization of echo streams, making it possible for multiple singers to avoid mutual interference. The model also proposes that the variety of units and unit patterns that singers produce result from multiple echo processing strategies being used in parallel. Understanding the perceptual processes that humpback whales bring to bear when listening to song-generated echo streams is critical to identifying those strategies.

The sonar model neither predicts nor explains why singers converge on similar song forms, nor does it account for why those forms progressively change over time. The model does suggest, however, that progressive changes in songs are non-arbitrary. Some bat species gradually transform their sonar signals over time based either on sounds they hear conspecifics producing (Moss et al., 2011; Furusawa et al., 2012), or on their familiarity with an environment (Wund, 2005; Chen et al., 2015), but none change their signals as extensively as humpback whales. Researchers have often interpreted collective changes in song structure as evidence that all singers are imitators and that some are also innovators. An alternative possibility, however, is that singers might select from a set of possible vocal adjustments based on what they hear happening in their surroundings, much like a fish within a school adjusts its swimming movements based on the movements of other nearby fish (Ballerini et al., 2008a; Cavagna et al., 2010). In this scenario, a singer conforms to other singers simply by selecting its actions within a shared auditory context. Like a dancer entering a club, where the sounds themselves cue the appropriate dance timing and style, a singer need not copy others or “learn how to dance” if a relevant repertoire of vocal acts is already available. When behavior is collective, simple rules of adjustment based on the actions of others can lead to complex, coherent dynamics (Ballerini et al., 2008b; Gorbonos et al., 2016). In general, as the number and density of singers within an area increase, the challenge of parsing the resulting auditory soundscape will increase. The sonar model thus predicts that the acoustic properties of songs within choruses should differ systematically from those of songs produced in less complicated contexts. For instance, the sonar model predicts that the acoustic properties of units, duration of themes/songs, or timing of units produced in foraging contexts should differ systematically from those produced on breeding grounds.

Winn and Winn (1978) were the first to note that song units might be functionally heterogeneous. Specifically, they hypothesized that “surface ratchets” might enable a singer to estimate its distance from the surface, that “moans and snores” (CF/qCF units) might be specialized for long-distance contact calling, and that more variable, shorter-range, broadband sounds might communicate more detailed information. The duplex sonar model similarly suggests that different types of sounds play different functional roles, as is the case in man-made sonar systems (Denny, 2007; Hague and Buck, 2017), with CF/qCF units maximizing target detectability and broadband/FM units facilitating target localization. By focusing on the echo-generating potential of units, the model accounts for why singers systematically transform units in predictable ways, why they are more likely to modulate spectral properties of units than temporal features, and why they maintain stable relationships between the acoustic features of sequential units. The model also predicts that singers will modulate unit properties depending on the acoustic conditions that they encounter. Unlike man-made sonar systems, humpback whales do not have the luxury of using large arrays of hydrophones to detect and localize targets. Humpback whales may make up for this lack of spatiotemporal resolution by customizing sounds so that echo streams can be interpreted using sparsely distributed cues (e.g., see Cooke, 2006), in which case the apparent complexity of songs may be a consequence of the perceptual challenges that humpback whales face when echolocating long distances underwater.

Humpback whales are flexible vocalizers that provide unique opportunities for studying echoic perception and spatial hearing more generally. They are slow moving, use sounds audible to humans, detect conspecifics at long distances, modulate their sounds over long time-frames, and produce thousands of sounds each day. Fifty years ago, many were incredulous that a whale might sing long, complicated songs. Today, the possibility that those same songs might enable humpback whales to perceive other whales from kilometers away may seem even more far-fetched. But, what should we expect an echolocating humpback whale to look or sound like? The sonar model provides a sketch of what to expect, and experimental tests of its predictions remain the best way to evaluate its utility and validity.

## Author Contributions

EM contributed the content of this manuscript.

## Conflict of Interest Statement

The author declares that the research was conducted in the absence of any commercial or financial relationships that could be construed as a potential conflict of interest.

## Footnotes

1 Although the time bandwidth product of a signal has a lower absolute limit that will necessarily cause very short duration signals to have a higher bandwidth, humpback whale units do not approach this limit. Singers sometimes produce 500 ms CF units as well as broadband units longer than 2 s, and in principle, unit duration could be positively correlated with bandwidth or uncorrelated.

## References

[B1] Adams A. M. Davis K. Smotherman M. (2017). Suppression of emission rates improves sonar performance by flying bats. *Sci. Rep.* 7:41641. 10.1038/srep41641 28139707 PMC5282581

[B2] Au W. W. L. Frankel A. S. Helweg D. A. Cato D. H. (2001). Against the humpback whale sonar hypothesis. *IEEE J. Ocean. Eng.* 26 295–300. 10.1109/48.922795

[B3] Au W. W. L. Mobley J. Burgess W. C. Lammers M. O. Nachitgall P. E. (2000). Seasonal and diurnal trends of chorusing humpback whales wintering in waters off western Maui. *Mar. Mamm. Sci.* 16 530–544. 10.1111/j.1748-7692.2000.tb00949.x

[B4] Au W. W. L. Pack A. A. Lammers M. O. Herman L. M. Deakos M. H. Andrews K. (2006). Acoustic properties of humpback whale songs. *J. Acoust. Soc. Am.* 120 1103–1110. 10.1121/1.2211547 16938996

[B5] Balcombe J. P. Fenton M. B. (1988). Eavesdropping by bats: the influence of echolocation call design and foraging strategy. *Ethology* 79 158–166. 10.1111/j.1439-0310.1988.tb00708.x

[B6] Ballerini M. Cabibbo N. Candelier R. Cavagna A. Cisbani E. Giardina I. (2008a). Empirical investigation of starling flocks: a benchmark study in collective animal behavior. *Anim. Behav.* 76 201–215. 10.1016/j.anbehav.2008.02.004

[B7] Ballerini M. Cabibbo N. Candelier R. Cavagna A. Cisbani E. Giardina I. (2008b). Interaction ruling animal collective behavior depends on topological rather than metric distance: evidence from a field study. *Proc. Natl. Acad. Sci. U.S.A.* 105 1232–1237. 10.1073/pnas.0711437105 18227508 PMC2234121

[B8] Bee M. A. Micheyl C. (2008). The cocktail problem: what is it? How can it be solved? And why should animal behaviorists study it? *J. Comp. Psychol.* 122 235–251. 10.1037/0735-7036.122.3.235 18729652 PMC2692487

[B9] Branstetter B. K. Mercado E. Au W. L. (2007). Representing multiple discrimination cues in a computational model of the bottlenose dolphin auditory system. *J. Acoust. Soci. Am.* 122 2459–2468. 10.1121/1.2772214 17902881

[B10] Branstetter B. K. Mercado E. III (2006). Sound localization by cetaceans. *Int. J. Comp. Psychol.* 19 26–61.

[B11] Brinklov S. Fenton M. B. Ratcliffe J. M. (2013). Echolocation in oilbirds and swiftlets. *Front. Physiol.* 4:123. 10.3389/fphys.2013.00123 23755019 PMC3664765

[B12] Caetano M. Rodet X. (2013). Musical instrument sound morphing guided by perceptually motivated features. *IEEE Trans. Audio Speech Lang. Process.* 21 1666–1675. 10.1109/TASL.2013.2260154

[B13] Catchpole C. K. (1987). Bird song, sexual selection and female choice. *Trends Ecol. Evol.* 2 94–97. 10.1016/0169-5347(87)90165-0 21227827

[B14] Catchpole C. K. Slater P. J. B. (2008). *Bird Song: Biological themes and Variations.* Cambridge: Cambridge University Press 10.1017/CBO9780511754791

[B15] Cato D. H. (1991). Songs of humpback whales: the Australian perspective. *Mem. Queensl. Mus.* 30 277–290.

[B16] Cavagna A. Cimarelli A. Giardina I. Parisi G. Santagati R. Stefanini F. (2010). From empirical data to inter-individual interactions: unveiling the rules of collective animal behavior. *Math. Models Methods Appl. Sci.* 20 1491–1510. 10.1142/S0218202510004660

[B17] Cazau D. Adam O. Aubin T. Laitman J. T. Reidenberg J. S. (2016). A study of vocal nonlinearities in humpback whale songs: from production mechanisms to acoustic analysis. *Sci. Rep.* 6:31660. 10.1038/srep31660 27721476 PMC5056341

[B18] Cazau D. Adam O. Laitman J. T. Reidenberg J. S. (2013). Understanding the intentional acoustic behavior of humpback whales: a production-based approach. *J. Acoust. Soc. Am.* 134 2268–2273. 10.1121/1.4816403 23967956

[B19] Cerchio S. Jacobsen J. K. Norris T. F. (2001). Temporal and geographical variations in songs of humpback whales, *Megaptera novaeangliae*: synchronous change in Hawaiian and Mexican breeding assemblages. *Anim. Behav.* 62 313–329. 10.1006/anbe.2001.1747

[B20] Chen Y. Liu Q. Shao Y. G. Tan L. J. Xiang Z. F. Zhang L. B. (2015). Variation in echolocation calls of *Hipposideros armiger* during habituation to a novel, captive environment. *Behaviour* 152 1083–1095. 10.1163/1568539X-00003269

[B21] Cholewiak D. Sousa-Lima R. Cerchio S. (2013). Humpback whale song hierarchical structure: historical context and discussion of current classification issues. *Mar. Mamm. Sci.* 29 E312–E332. 10.1111/mms.12005

[B22] Cholewiak D. M. Cerchio S. Jacobsen J. K. Urban-R J. Clark C. W. (2018). Songbird dynamics under the sea: acoustic interactions between humpback whales suggest song mediates male interactions. *R. Soc. Open Sci.* 5:171298. 10.1098/rsos.171298 29515847 PMC5830736

[B23] Clark C. W. Clapham P. J. (2004). Acoustic monitoring on a humpback whale (*Megaptera novaeangliae*) feeding ground shows continual singing into late Spring. *Proc. R. Soc. Lond. B* 271 1051–1057. 10.1098/rspb.2004.2699 15293859 PMC1691688

[B24] Cooke M. (2006). A glimpsing model of speech perception in noise. *J. Acoust. Soc. Am.* 119 1562–1573. 10.1121/1.2166600 16583901

[B25] Darling J. D. (1983). *Migration, Abundance and Behavior of Hawaiian Humpback Whales (Megaptera novaeangliae).* Doctoral dissertation, University of California, Santa Cruz, CA.

[B26] Darling J. D. Berube M. (2001). Interactions of singing humpback whales with other males. *Mar. Mamm. Sci.* 17 570–584. 10.1111/j.1748-7692.2001.tb01005.x

[B27] Darling J. D. Jones M. E. Nicklin C. P. (2012). Humpback whale (*Megaptera novaeangliae*) singers in Hawaii are attracted to playback of similar song (L). *J. Acoust. Soc. Am.* 132 2955–2958. 10.1121/1.4757739 23145581

[B28] Darling J. D. Meagan E. Nicklin C. P. (2006). Humpback whale songs: do they organize males during the breeding season? *Behaviour* 143 1051–1101. 10.1163/156853906778607381

[B29] Darwin C. R. (1871). *The Descent of Man, and Selection in Relation to Sex.* London: John Murray.

[B30] Decarpigny J.-N. Hamonic B. Wilson O. B. (1991). The design of low-frequency underwater acoustic projectors: present status and future trends. *IEEE J. Ocean. Eng.* 16 107–121. 10.1109/48.64890

[B31] Denny M. (2007). *Blip, Ping, and Buzz: Making Sense of Radar and Sonar.* Baltimore, MD: John Hopkins University Press.

[B32] Dunlop R. A. (2016). Changes in vocal parameters with social context in humpback whales: considering the effect of bystanders. *Behav. Ecol. Sociobiol.* 70 857–870. 10.1007/s00265-016-2108-0 27217614 PMC4859862

[B33] Dunlop R. A. Noad M. J. (2016). The “risky” business of singing: tactical use of song during joining by male humpback whales. *Behav. Ecol. Sociobiol.* 70 2149–2160. 10.1007/s00265-016-2218-8

[B34] Edds-Walton P. L. (1997). Acoustic communication signals of mysticete whales. *Bioacoustics* 8 47–60. 10.1080/09524622.1997.9753353

[B35] Ellis D. P. W. (2009). *Gammatone**-Like Spectrograms* Available at: www.ee.columbia.edu/ln/rosa/matlab/gammatonegram

[B36] Ellison W. T. Clark C. W. Bishop G. C. (1987). Potential use of surface reverberation by bowhead whales, *Balaena mysticetus*, in under-ice navigation. *Rep. Int. Whal. Comm.* 37 329–332.

[B37] Escera C. Alho K. Winkler I. Naatanen R. (1998). Neural mechanisms of involuntary attention to acoustic novelty and change. *J. Cogn. Neurosci.* 10 590–604. 10.1162/089892998562997 9802992

[B38] Español-Jiménez S. van der Schaar M. (2018). First record of humpback whale songs in Southern Chile: analysis of seasonal and diel variation. *Mar. Mamm. Sci.* 10.1111/mms.12477 [Epub ahead of print].

[B39] Fawcett K. Ratcliffe J. M. (2015). Clutter and conspecifics: a comparison of their influence on echolocation and flight behaviour in Daubenton’s bat, *Myotis daubentonii*. *J. Comp. Physiol. A Neuroethol. Sens. Neural. Behav. Physiol.* 201 295–304. 10.1007/s00359-014-0977-0 25552318

[B40] Feekes F. (1982). Song mimesis within colonies of *Cacicus c. cela (Icteridae, Aves)*: a colonial password? *Ethology* 58 119–152. 10.1111/j.1439-0310.1982.tb00312.x

[B41] Feng L. Oxenham A. J. (2015). New perspectives on the measurement and time course of auditory enhancement. *J. Exp. Psychol. Hum. Percept. Perform.* 41 1696–1708. 10.1037/xhp0000115 26280269 PMC4666811

[B42] Fenton M. B. (2003). Eavesdropping on the echolocation and social calls of bats. *Mamm. Rev.* 33 193–204. 10.1046/j.1365-2907.2003.00019.x

[B43] Fenton M. B. Faure P. A. Ratcliffe J. M. (2012). Evolution of high duty cycle echolocation in bats. *J. Exp. Biol.* 215 2935–2944. 10.1242/jeb.073171 22875762

[B44] Fenton M. B. Jensen F. B. Kalko E. K. Tyack P. L. (2014). “Sonar signals of bats and toothed whales,” in *Biosonar*, eds Surlykke A. Nachitgall P. E. Fay R. R. Popper A. N. (New York, NY: Springer), 11–60.

[B45] Finneran J. J. (2013). Dolphin “packet” use during long-range echolocation tasks. *J. Acoust. Soc. Am.* 133 1796–1810. 10.1121/1.4788997 23464048

[B46] Finneran J. J. Schroth-Miller M. Borror N. Tormey M. Brewer A. Black A. (2014). Multi-echo processing by a bottlenose dolphin operating in “packet” transmission mode at long range. *J. Acoust. Soc. Am.* 136 2876–2886. 10.1121/1.4898043 25373986

[B47] Fordyce R. E. Barnes L. G. (1994). The evolutionary history of whales and dolphins. *Annu. Rev. Earth Planet. Sci.* 22 419–455. 10.1146/annurev.ea.22.050194.002223

[B48] Frankel A. S. Clark C. W. Herman L. M. Gabriele C. M. (1995). Spatial distribution, habitat utilization, and social interactions of humpback whales, *Megaptera novaeangliae*, off Hawai’i, determined using acoustic and visual techniques. *Can. J. Zool.* 73 1134–1146. 10.1139/z95-135

[B49] Frazer L. N. Mercado E. I. I. I. (2000). A sonar model for humpback whale song. *IEEE J. Ocean. Eng.* 25 160–182. 10.1109/48.820748

[B50] Fristrup K. M. Hatch L. T. Clark C. W. (2003). Variation in humpback whale (*Megaptera novaeangliae*) song length in relation to low-frequency sound broadcasts. *J. Acoust. Soc. Am.* 113 3411–3424. 10.1121/1.1573637 12822811

[B51] Furusawa Y. Hiryu S. Kobayasi K. I. Riquimaroux H. (2012). Convergence of reference frequencies by multiple CF-FM bats (*Rhinolophus ferrumequinum nippon*) during paired flights evaluated with onboard microphones. *J. Comp. Physiol. A Neuroethol. Sens. Neural. Behav. Physiol.* 198 683–693. 10.1007/s00359-012-0739-9 22717760

[B52] Garland E. C. Gedamke J. Rekdahl M. L. Noad M. J. Garrigue C. Gales N. (2013). Humpback whale song on the Southern Ocean feeding grounds: implications for cultural transmission. *PLoS One* 8:e79422. 10.1371/journal.pone.0079422 24278134 PMC3835899

[B53] Garland E. C. Goldizen A. W. Rekdahl M. L. Constantine R. Garrigue C. Hauser N. D. (2011). Dynamic horizontal cultural transmission of humpback whale song at the ocean basin scale. *Curr. Biol.* 21 687–691. 10.1016/j.cub.2011.03.019 21497089

[B54] Garland E. C. Rendell L. Lamoni L. Poole M. M. Noad M. J. (2017). Song hybridization events during revolutionary song change provide insights into cultural transmission in humpback whales. *Proc. Natl. Acad. Sci. U.S.A.* 114 7822–7829. 10.1073/pnas.1621072114 28739940 PMC5543391

[B55] Geist V. (1971). *Mountain Sheep: A Study in Behavior and Evolution.* Chicago, IL: University of Chicago Press.

[B56] George J. C. Clark C. W. Carroll G. M. Ellison W. T. (1988). Observations on the ice-breaking and ice navigation behavior of migrating bowhead whales (*Balaena mysticetus*) near Point Barrow, Alaska, Spring 1985. *Arctic* 42 24–30.

[B57] Geva-Sagiv M. Las L. Yovel Y. Ulanovsky N. (2015). Spatial cognition in bats and rats: from sensory acquisition to multiscale maps and navigation. *Nat. Rev. Neurosci.* 16 94–108. 10.1038/nrn3888 25601780

[B58] Gong Z. Jain A. D. Tran D. Yi D. H. Wu F. Zorn A. (2014). Ecosystem scale acoustic sensing reveals humpback whale behavior synchronous with herring spawning processes and re-evaluation finds no effect of sonar on humpback song occurrence in the Gulf of Maine in fall 2006. *PLoS One* 9:e104733. 10.1371/journal.pone.0104733 25289938 PMC4188555

[B59] Gorbonos D. Ianconescu R. Puckett J. G. Ni R. Ouellette N. T. Gov N. S. (2016). Long-range acoustic interactions in insect swarms: an adaptive gravity model. *New J. Phys.* 18:073042 10.1088/1367-2630/18/7/073042

[B60] Gray P. M. Krause B. Atema J. Payne R. Krumhansl C. Baptista L. (2001). Biology and music. *Music Nat. Sci.* 291 52–54. 10.1126/science.10.1126/science.1056960 11192008

[B61] Green S. Marler P. (1979). “The analysis of animal communication,” in *Handbook of Behavioral Neurobiology 3: Social Behavior and Communication*, eds Marler P. Vandenbergh J. G. (New York, NY: Plenum Press),73–158.

[B62] Green S. R. Mercado E. III Pack A. A. Herman L. M. (2011). Recurring patterns in the songs of humpback whales (*Megaptera novaeangliae*). *Behav. Process.* 86 284–294. 10.1016/j.beproc.2010.12.014 21215306

[B63] Griffin D. R. (1958). *Listening in the Dark: The Acoustic Orientation of Bats and Men.* Oxford: Yale University Press.

[B64] Guinee L. N. Payne K. B. (1988). Rhyme-like repetitions in songs of humpback whales. *Ethology* 79 295–306. 10.1111/j.1439-0310.1988.tb00718.x 19507926

[B65] Hague D. A. Buck J. R. (2017). The generalized sinusoidal frequency-modulated waveform for active sonar. *IEEE J. Ocean. Eng.* 42 109–123. 10.1121/1.5113581 31255136

[B66] Handel S. Todd S. K. Zoidis A. M. (2012). Hierarchical and rhythmic organization in the songs of humpback whales (*Megaptera novaeangliae*). *Bioacoustics* 21 141–156. 10.1121/1.3124712 19507926

[B67] Hase K. Kadoya Y. Maitani Y. Miyamoto T. Kobayasi K. I. Hiryu S. (2018). Bats enhance their call identities to solve the cocktail party problem. *Commun. Biol.* 1:39 10.1038/s42003-018-0045-3 PMC6123623 30271924

[B68] Helweg D. A. Frankel A. S. Mobley J. Herman L. M. (1992). “Humpback whale song: our current understanding,” in *Marine Mammal Sensory Systems*, eds Thomas J. A. Kastelein R. A. Supin A. S. (New York, NY: Plenum), 459–483. 10.1007/978-1-4615-3406-8_32

[B69] Henderson E. E. Helble T. A. Ierley G. R. Martin S. W. (2018). Identifying behavioral states and habitat use of acoustically tracked humpback whales in Hawaii. *Mar. Mamm. Sci.* 10.1111/mms.12475 [Epub ahead of print].

[B70] Henson O. W. (1987). Biosonar imaging of insects by *Pteronotus p. parnelli*, the mustached bat. *Natl. Geogr. Res.* 3 82–101.

[B71] Herman L. M. (2017). The multiple functions of male song within the humpback whale (*Megaptera novaeangliae*) mating system: review, evaluation, and synthesis. *Biol. Rev.* 92 1795–1818. 10.1111/brv.12309 28677337

[B72] Herman L. M. Tavolga W. N. (1980). “The communication systems of cetaceans,” in *Cetacean Behavior: Mechanisms and Functions*, ed. Herman L. M. (New York, NY: Wiley Interscience), 149–209.

[B73] Huang W. Wang D. Ratilal P. (2016). Diel and spatial dependence of humpback song and non-song vocalizations in fish spawning ground. *Remote Sens.* 8:712. 10.1038/nature16960 26934221

[B74] Hulse S. H. (2002). Auditory scene analysis in animal communication. *Adv. Study Behav.* 31 163–200. 10.1016/S0065-3454(02)80008-0

[B75] Janik V. M. (2009). Whale song. *Curr. Biol.* 19 R109–R111. 10.1016/j.cub.2008.11.026 19211045

[B76] Jung K. Kalko E. K. V. Helversen O. V. (2007). Echolocation calls in Central American emballonurid bats: signal design and call frequency alternation. *J. Zool.* 272 125–137. 10.1111/j.1469-7998.2006.00250.x

[B77] Ketten D. R. (1992). “The marine mammal ear: Specializations for aquatic conditions and echolocation,” in *The Evolutionary Biology of Hearing*, eds Webster D. B. Fay R. R. Popper A. N. (New York, NY: Springer),717–750.

[B78] Ketten D. R. (1997). Structure and function in whale ears. *Bioacoustics* 8 103–135. 10.1080/09524622.1997.9753356

[B79] Kowarski K. Evers C. Moors-Murphy H. Martin B. Denes S. L. (2018). Singing through winter nights: seasonal and diel occurence of humpback whale (*Megaptera novaeangliae*) calls in and around the Gully MPA, offshore eastern Canada. *Mar. Mamm. Sci.* 34 169–189. 10.1111/mms.12447

[B80] LePage K. D. (1998). *Bottom Reverberation in Shallow Water: Coherent Properties as a Function of Bandwidth, Waveguide, Characteristics and Scatterer Distributions.* La Spezia: SACLANT Undersea Research Centre SR, 301.

[B81] Lewicki M. S. Olshausen B. A. Surlykke A. Moss C. F. (2014). Scene analysis in the natural environment. *Front. Psychol.* 5:199. 10.3389/fpsyg.2014.00199 24744740 PMC3978336

[B82] MacKay M. M. Wursig B. Bacon C. E. Selwayn J. D. (2016). North Atlantic humpback whale (*Megaptera novaeangliae*) hotspots defined by bathymetric features off western Puerto Rico. *Can. J. Zool.* 94 517–527. 10.1139/cjz-2015-0198

[B83] Manning J. T. (1985). Choosy females and correlates of male age. *J. Theor. Biol.* 116 349–354. 10.1016/S0022-5193(85)80273-3

[B84] Mercado E. III Frazer L. N. (1999). Environmental constraints on sound transmission by humpback whales. *J. Acoust. Soc. Am.* 106 3004–3016. 10.1121/1.428120 10573910

[B85] Mercado E. III Frazer L. N. (2001). Humpback whale song or humpback whale sonar? A reply to Au *IEEE J. Ocean. Eng.* 26 406–415. 10.1109/48.946514

[B86] Mercado E. III Green S. R. Schneider J. N. (2008). Understanding auditory distance estimation by humpback whales: a computational approach. *Behav. Process.* 77 231–242. 10.1016/j.beproc.2007.10.007 18068910

[B87] Mercado E. III Handel S. (2012). Understanding the structure of humpback whale songs (L). *J. Acoust. Soc. Am.* 132 2947–2950. 10.1121/1.4757643 23145579

[B88] Mercado E. III Herman L. M. Pack A. A. (2003). Stereotypical sound patterns in humpback whale songs: usage and function. *Aqu. Mamm.* 29 37–52. 10.1578/016754203101024068

[B89] Mercado E. III Herman L. M. Pack A. A. (2005). Song copying by humpback whales: themes and variations. *Anim. Cogn.* 8 93–102. 10.1007/s10071-004-0238-7 15490289

[B90] Mercado E. III Mantell J. T. Pfordresher P. Q. (2014). Imitating sounds: a cognitive approach to understanding vocal imitation. *Comp. Cogn. Behav. Rev.* 9 1–57. 10.3819/ccbr.2014.90002

[B91] Mercado E. III Schneider J. N. Pack A. A. Herman L. M. (2010). Sound production by singing humpback whales. *J. Acoust. Soc. Am.* 127 2678–2691. 10.1121/1.3309453 20370048

[B92] Mercado E. III Sturdy C. B. (2017). “Classifying animal sounds with neural networks,” in *Comparative Bioacoustics: An Overview*, eds Brown C. Riede T. (Sharjah: Bentham Science), 415–461.

[B93] Mercado E. I. I. I. (2016). Acoustic signaling by singing humpback whales (*Megaptera novaeangliae*): what role does reverberation play? *PLoS One* 11:e0167277. 10.1371/journal.pone.0167277 27907182 PMC5132011

[B94] Miller P. J. O. Biassoni N. Samuels A. Tyack P. L. (2000). Whale songs lengthen in response to sonar. *Nature* 405:903. 10.1038/35016148 10879521

[B95] Mobley J. R. Jr. Herman L. M. Frankel A. S. (1988). Responses of wintering humpback whales (*Megaptera novaeangliae*) to playback of recordings of winter and summer vocalizations and of synthetic sound. *Behav. Ecol. Sociobiol.* 23 211–223. 10.1007/BF00302944

[B96] Mora E. C. IbaìÑEz C. Macias S. Juste J. Lopez I. Torres L. (2011). Plasticity in the echolocation inventory of *Mormopterus minutus* (Chiroptera, Molossidae). *Acta Chiropterol.* 13 179–187. 10.3161/150811011X578723

[B97] Moss C. F. Chiu C. Moore P. M. (2014). “Analysis of natural scenes by echolocation in bats and dolphins,” in *Biosonar*, eds Surlykke A. Nachitgall P. E. Fay R. R. Popper A. N. (New York, NY: Springer), 231–256.

[B98] Moss C. F. Chiu C. Surlykke A. (2011). Adaptive vocal behavior drives perception by echolocation in bats. *Curr. Opin. Neurobiol.* 21 645–652. 10.1016/j.conb.2011.05.028 21705213 PMC3178000

[B99] Moss C. F. Surlykke A. (2010). Probing the natural scene by echolocation in bats. *Front. Behav. Neurosci.* 4:33. 10.3389/fnbeh.2010.00033 20740076 PMC2927269

[B100] Mruczek R. E. Sheinberg D. L. (2005). Distractor familiarity leads to more efficient visual search for complex stimuli. *Percept. Psychophys.* 67 1016–1031. 10.3758/BF03193628 16396010

[B101] Muller R. Schnitzler H. U. (2000). Acoustic flow perception in cf-bats: extraction of parameters. *J. Acoust. Soc. Am.* 108 1298–1307. 10.1121/1.1287842 11008830

[B102] Naatanen R. Paavilainen P. Rinne T. Alho K. (2007). The mismatch negativity (MMN) in basic research of central auditory processing: a review. *Clin. Neurophysiol.* 118 2544–2590. 10.1016/j.clinph.2007.04.026 17931964

[B103] Naguib M. Riebel K. (2014). “Singing in space and time: the biology of birdsong,” in *Biocommunication of Animals*, ed. Witzany G. (Dordrecht: Springer), 233–247. 10.1007/978-94-007-7414-8_13

[B104] Noad M. J. Cato D. H. Bryden M. M. Jenner M. N. Jenner K. C. (2000). Cultural revolution in whale songs. *Nature* 408:537. 10.1038/35046199 11117730

[B105] Noad M. J. Cato D. H. Stokes M. D. (2004). “Acoustic tracking of humpback whales: measuring interactions with the acoustic environment,” in *Proceedings of the Annual Conference of the Australian Acoustical Society, Gold Coast*, Castlemaine, 353–358.

[B106] Norris K. S. (1966). “Some observations on the migration and orientation of marine mammals,” in *Animal Orientation and Navigation*, ed. Storm R. M. (Corvallis, OR: Oregon State University Press), 101–125.

[B107] Obrist M. K. (1995). Flexible bat echolocation: the influence of individual habitat and conspecifics on sonar signal design. *Behav. Ecol. Sociobiol.* 36 207–219. 10.1007/BF00177798

[B108] Odom K. J. Hall M. L. Riebel K. Omland K. E. Langmore N. E. (2014). Female song is widespread and ancestral in songbirds. *Nat. Commun.* 5:3379. 10.1038/ncomms4379 24594930

[B109] Ou H. Au W. W. Zurk L. M. Lammers M. O. (2013). Automated extraction and classification of time-frequency contours in humpback vocalizations. *J. Acoust. Soc. Am.* 133 301–310. 10.1121/1.4770251 23297903

[B110] Parsons E. C. M. Wright A. J. Gore M. A. (2008). The nature of humpback whale (*Megaptera novaeangliae*) song. *J. Mar. Anim. Their Ecol.* 1 22–31. 23039423

[B111] Payne K. (2000). “The progressively changing songs of humpback whales: a window on the creative process in a wild animal,” in *Origins of Music*, eds Wallin N. L. Merker B. Brown S. (Cambridge, MA: MIT Press), 135–150.

[B112] Payne K. Payne R. S. (1985). Large scale changes over 19 years in songs of humpback whales in Bermuda. *Z. Tierpsychol.* 68 89–114. 10.1111/j.1439-0310.1985.tb00118.x

[B113] Payne K. Tyack P. Payne R. S. (1983). “Progressive changes in the songs of humpback whales (*Megaptera novaeangliae*): a detailed analysis of two seasons in Hawaii,” in *Communication and Behavior of Whales*, ed. Payne R. (Boulder, CO: Westview Press), 9–57.

[B114] Payne R. B. (1985). Behavioral continuity and change in local song populations of village indigobirds. *Z. Tierpsychol.* 70 1–44. 10.1111/j.1439-0310.1985.tb00498.x 564106

[B115] Payne R. S. McVay S. (1971). Songs of humpback whales. *Science* 173 585–597. 10.1126/science.173.3997.585 17833100

[B116] Ratcliffe J. M. Jakobsen L. Kalko E. K. Surlykke A. (2011). Frequency alternation and an offbeat rhythm indicate foraging behavior in the echolocating bat, *Saccopteryx bilineata*. *J. Comp. Physiol. A Neuroethol. Sens. Neural. Behav. Physiol.* 197 413–423. 10.1007/s00359-011-0630-0 21327333

[B117] Reicher G. M. (1976). Familiarity of background characters in visual scanning. *J. Exp. Psychol. Hum. Percept. Perform.* 2 522–530. 10.1037/0096-1523.2.4.522 1011001

[B118] Richards J. T. Reicher G. M. (1978). The effect of background familiarity in visual search: an analysis of underlying factors. *Percept. Psychophys.* 23 499–505. 10.3758/BF03199526

[B119] Rothenberg D. (2014). *Whale Song Explained.* Available at: https://medium.com/@dealville/whales-synchronize-their-songs-across-oceans-and-theres-sheet-music-to-prove-it-b1667f603844 [accessed May 25 2018].

[B120] Rothenberg D. (2015). *Maui One.* On *New Songs of the Humpback Whale.* Groveland, MA: Important Records.

[B121] Sardelis S. (2017). *Why do Whales Sing?* Available at: https://www.youtube.com/watch?v=7Xr9BYhlceA [accessed October 20 2018].

[B122] Schneider J. N. Lloyd D. R. Banks P. N. Mercado E. I. I. I. (2014). Modeling the utility of binaural cues for underwater sound localization. *Hear. Res.* 312 103–113. 10.1016/j.heares.2014.03.011 24727491

[B123] Schneider J. N. Mercado E. III (2018). Characterizing the rhythm and tempo of sound production by singing whales. *Bioacoustics* 10.1080/09524622.2018.1428827

[B124] Searcy W. A. Andersson M. (1986). Sexual selection and the evolution of song. *Annu. Rev. Ecol. Syst.* 17 507–533. 10.1146/annurev.es.17.110186.002451

[B125] Seger K. D. Thode A. M. Urban-R J. Martinez-Loustalot P. Jimenez-Lopez M. E. Lopez-Arzate D. (2016). Humpback whale-generated ambient noise levels provide insight into singers’ spatial densities. *J. Acoust. Soc. Am.* 140 1581–1597. 10.1121/1.4962217 27914437

[B126] Simmons J. A. (1989). A view of the world through the bat’s ear: the formation of acoustic images in echolocation. *Cognition* 33 155–199. 10.1016/0010-0277(89)90009-7 2691182

[B127] Simmons J. A. Eastman K. M. Auger G. O’farrell M. J. Grinnell A. D. Griffin D. R. (2004). Video/acoustic-array studies of swarming by echolocating bats. *J. Acoust. Soc. Am.* 116:2632 10.1121/1.4785500

[B128] Simmons J. A. Houser D. Kloepper L. (2014). “Localization and classification of targets by echolocating bats and dolphins,” in *Biosonar*, eds Surlykke A. Nachitgall P. E. Fay R. R. Popper A. N. (New York, NY: Springer), 169–194.

[B129] Simmons J. A. Stein R. A. (1980). Acoustic imaging in bat sonar: echolocation signals and the evolution of echolocation. *J. Comp. Physiol.* 135 61–84. 10.1007/BF00660182

[B130] Smith J. N. Goldizen A. W. Dunlop R. A. Noad M. J. (2008). Songs of male humpback whales, *Megaptera novaeangliae*, are involved in intersexual interactions. *Anim. Behav.* 76 467–477. 10.1016/j.anbehav.2008.02.013

[B131] Sousa-Lima R. (2007). *Acoustic Ecology of Humpback Whales (Megaptera novaeangliae) in the Abrolhos National Marine Park, Brazil.* Doctoral dissertation, Cornell University, Ithaca, NY.

[B132] Spector D. A. (1994). Definition in biology: the case of “bird song”. *J. Theor. Biol.* 168 373–381. 10.1006/jtbi.1994.1117

[B133] Stanistreet J. E. Risch D. Van Parijs S. M. (2013). Passive acoustic tracking of singing humpback whales (*Megaptera novaeangliae*) on a northwest Atlantic feeding ground. *PLoS One* 8:e61263. 10.1371/journal.pone.0061263 23593447 PMC3622601

[B134] Stimpert A. K. Peavey L. E. Friedlaender A. S. Nowacek D. P. (2012). Humpback whale song and foraging behavior on an Antarctic feeding ground. *PLoS One* 7:e51214. 10.1371/journal.pone.0051214 23284666 PMC3526533

[B135] Stimpert A. K. Wiley D. N. Au W. W. Johnson M. P. Arsenault R. (2007). ‘Megapclicks’: acoustic click trains and buzzes produced during night-time foraging of humpback whales (*Megaptera novaeangliae*). *Biol. Lett.* 3 467–470. 10.1098/rsbl.2007.0281 17686753 PMC2391189

[B136] Surlykke A. Nachitgall P. E. Fay R. R. Popper A. N. (eds) (2014). *Biosonar.* New York, NY: Springer 10.1007/978-1-4614-9146-0

[B137] Tobias J. A. Seddon N. (2009). Signal jamming mediates sexual conflict in a duetting bird. *Curr. Biol.* 19 577–582. 10.1016/j.cub.2009.02.036 19285404

[B138] Tyack P. L. (1981). Interactions between singing Hawaiian humpback whales and conspecifics nearby. *Behav. Ecol. Sociobiol.* 8 105–116. 10.1007/BF00300822

[B139] Tyack P. L. (1997). Studying how cetaceans use sound to explore their environment. *Perspect. Ethol.* 12 251–297. 10.1242/jeb.151498 28883086

[B140] Tyack P. L. Clark C. W. (2000). “Communication and acoustic behavior of dolphins and whales,” in *Hearing by Whales and Dolphins*, eds Au W. W. L. Popper A. N. Fay R. R. (New York, NY: Springer), 156–224s. 10.1007/978-1-4612-1150-1_4

[B141] Van Olst E. H. (1971). *The Orienting Reflex.* Dordrecht: The Hague.

[B142] Vu E. T. Risch D. Clark C. W. Gaylord S. Hatch L. T. Thompson M. A. (2012). Humpback whale song occurs extensively on feeding grounds in the western North Atlantic Ocean. *Aqu. Biol.* 14 175–183. 10.3354/ab00390

[B143] Wahlberg M. Surlykke A. (2014). “Sound intensities of biosonar signals from bats and toothed whales,” in *Biosonar*, eds Surlykke A. Nachitgall P. E. Fay R. R. Popper A. N. (New York, NY: Springer), 107–142. 10.1007/978-1-4614-9146-0_4

[B144] Wang D. Garcia H. Huang W. Tran D. D. Jain A. D. Yi D. H. (2016). Vast assembly of vocal marine mammals from diverse species on fish spawning ground. *Nature* 531 366–370. 10.1038/nature16960 26934221

[B145] Wang Q. Cavanagh P. Green M. (1994). Familiarity and pop-out in visual search. *Percept. Psychophys.* 56 495–500. 10.3758/BF03206946 7991347

[B146] Warnecke M. Chiu C. Engelberg J. Moss C. F. (2015). Active listening in a bat cocktail party: adaptive echolocation and flight behaviors of big brown bats, *Eptesicus fuscus*, foraging in a cluttered acoustic environment. *Brain Behav. Evol.* 86 6–16. 10.1159/000437346 26398707

[B147] Watkins W. A. George J. E. Daher M. A. Mullin D. L. Martin S. Haga H. (2000). *Whale Call Data for the North Pacific, November 1995 through July 1999, Occurrence of Calling Whales and Source Locations from SOSUS and other Acoustic Systems, WHOI-00-02.* Woods Hole, MA: Woods Hole Oceanographic Institute 10.21236/ADA375142

[B148] Willisch C. S. Neuhaus P. (2009). Alternative mating tactics and their impact on survival in the adult male alpine ibex (Capra Ibex Ibex). *J. Mammal.* 90 1421–1430. 10.1644/08-MAMM-A-316R1.1

[B149] Wilson E. O. (1975). *Sociobiology: The New Synthesis.* Cambridge, MA: Belknap Press.

[B150] Winn H. E. Perkins P. J. Poulter T. C. (1970). “Sounds of the humpback whale,” in *Proceedings of the 7th Annual Conference on Biological Sonar and Diving Mammals*, (Menlo Park, CA: Stanford Research Institute).

[B151] Winn H. E. Winn L. K. (1978). The song of the humpback whale *Megaptera novaeangliae* in the West Indies. *Mar. Biol.* 47 97–114. 10.1121/1.3309453 20370048

[B152] Wright A. J. Walsh L. A. (2010). Mind the gap: why neurological plasticity may explain seasonal interruption in humpback whale song. *J. Mar. Biol. Assoc. United Kingdom* 90 1489–1491. 10.1017/S0025315410000913

[B153] Wund M. A. (2005). Learning and the development of habitat-specific bat echolocation. *Anim. Behav.* 70 441–450. 10.1016/j.anbehav.2004.11.009

[B154] Yi D. H. Makris N. C. (2016). Feasibility of acoustic remote sensing of large herring shoals and seafloor by baleen whales. *Remote Sens.* 8:693 10.3390/rs8090693

[B155] Zhang Z. White P. R. (2017). A blind source separation approach for humpback whale song separation. *J. Acoust. Soc. Am.* 141 2705–2714. 10.1121/1.4980856 28464617

